# The MraY Inhibitor Muraymycin D2 and Its Derivatives Induce Enlarged Cells in Obligate Intracellular *Chlamydia* and *Wolbachia* and Break the Persistence Phenotype in *Chlamydia*

**DOI:** 10.3390/antibiotics13050421

**Published:** 2024-05-04

**Authors:** Iris Löckener, Lara Vanessa Behrmann, Jula Reuter, Andrea Schiefer, Anna Klöckner, Sebastian Krannich, Christian Otten, Katja Mölleken, Satoshi Ichikawa, Achim Hoerauf, Tanja Schneider, Kenneth M. Pfarr, Beate Henrichfreise

**Affiliations:** 1 Institute for Pharmaceutical Microbiology (IPM), University of Bonn, University Hospital Bonn, Meckenheimer Allee 168, 53115 Bonn, Germanycfotten@web.de (C.O.); bhenrich@uni-bonn.de (B.H.); 2 Institute for Medical Microbiology, Immunology and Parasitology (IMMIP), University Hospital Bonn, Venusberg-Campus 1, 53127 Bonn, Germany; lara_vanessa.behrmann@ukbonn.de (L.V.B.);; 3 Institute for Functional Microbial Genomics, Heinrich Heine University Düsseldorf, Universitätsstraße 1, 40225 Düsseldorf, Germany; katja.moelleken@hhu.de; 4 Faculty of Pharmaceutical Sciences, Hokkaido University, Kita-12, Nishi-6, Kita-ku, Sapporo 060-0812, Japan; 5 German Center for Infection Research (DZIF), Partner Site Bonn-Cologne, 53127 Bonn, Germany

**Keywords:** intracellular bacteria, *Chlamydia*, *Wolbachia*, cell division, peptidoglycan, lipid II synthesis, muraymycin, MraY, persistence-breaking

## Abstract

Chlamydial infections and diseases caused by filarial nematodes are global health concerns. However, treatment presents challenges due to treatment failures potentially caused by persisting *Chlamydia* and long regimens against filarial infections accompanied by low compliance. A new treatment strategy could be the targeting of the reduced peptidoglycan structures involved in cell division in the obligate intracellular bacteria *Chlamydia* and *Wolbachia*, the latter being obligate endosymbionts supporting filarial development, growth, and survival. Here, cell culture experiments with *C. trachomatis* and *Wolbachia* showed that the nucleoside antibiotics muraymycin and carbacaprazamycin interfere with bacterial cell division and induce enlarged, aberrant cells resembling the penicillin-induced persistence phenotype in *Chlamydia.* Enzymatic inhibition experiments with purified *C. pneumoniae* MraY revealed that muraymycin derivatives abolish the synthesis of the peptidoglycan precursor lipid I. Comparative in silico analyses of chlamydial and wolbachial MraY with the corresponding well-characterized enzyme in *Aquifex aeolicus* revealed a high degree of conservation, providing evidence for a similar mode of inhibition. Muraymycin D2 treatment eradicated persisting non-dividing *C. trachomatis* cells from an established penicillin-induced persistent infection. This finding indicates that nucleoside antibiotics may have additional properties that can break bacterial persistence.

## 1. Introduction

Bacteria of the genera *Chlamydia* and *Wolbachia* are obligate intracellular bacteria of medical importance. *Chlamydia* spp. are pathogens that undergo a unique biphasic developmental cycle within human epithelial and endothelial cells, whereas *Wolbachia* spp. reside in filarial nematodes and can cause diseases in humans.

*Chlamydia* infect host cells as condensed and metabolically less active elementary bodies (EBs). After invagination into the host cell, the EBs differentiate into non-infectious, metabolically active reticulate bodies (RBs) and replicate in the chlamydial inclusion. They asynchronously differentiate back into EBs and are released by host cell lysis or extrusion. One of the most relevant chlamydial pathogens is *C. trachomatis*. Serovars A-C cause ocular infections that are the leading cause of preventable blindness worldwide [1], while serovars D-K lead to ano-urogenital infections, and serovars L1-L3 cause lymphogranuloma venereum. The latter two are sexually transmitted infections. In 2019, 434,184 confirmed cases of genital chlamydial infection were recorded in member states of the European Union/European Economic Area [2]. Infections such as cervicitis in women and urethritis in men are often asymptomatic, but chronic or untreated chlamydial infections can have detrimental consequences for women, e.g., pelvic inflammatory disease, ectopic pregnancy, and miscarriage [3,4]. Currently, the WHO recommends doxycycline (DOX) or azithromycin (AZI) treatment for uncomplicated genital *Chlamydia* infections [5]. However, high rates of reoccurring chlamydial infections after treatment have been reported, ranging from a median of 13% in a meta-study to 31% for females in the USA [6,7,8]. About 8% of treatment failures cannot be attributed to reinfection by sexual partners [8,9].

Besides the possibility of reinfection from the gastro-intestinal tract [10], persistent *C. trachomatis* infection should be taken into consideration, as enlarged chlamydial cells could be observed in murine tissue under amoxicillin treatment [11] and in tissue from patients [6,12]. These enlarged and non-dividing cells, referred to as aberrant bodies (ABs), can be found in cell culture in the presence of stressors such as β-lactams and interferon-γ [13,14]. ABs are less metabolically active, persist within the host cell, and can re-enter the developmental cycle after the removal of the stressor [14,15]. Persisting chlamydial cells, including those induced by penicillin (PEN) treatment, are less susceptible to DOX and AZI in cell culture and in murine infection models [11,16,17,18], a phenomenon that may also contribute to treatment failure with these front-line antibiotics in patients.

*Wolbachia* spp. can be found in a range of arthropods and filarial nematodes [19,20,21]. In arthropods, these endobacteria are mostly facultative symbionts [22], whereas in the filarial nematodes *Wuchereria bancrofti*, *Brugia malayi*, *Brugia timori*, and *Onchocerca volvulus*, they are obligate endosymbionts that support filarial development and survival [23,24]. Filarial infections cause the neglected tropical diseases lymphatic filariasis, transmitted by mosquitos, and onchocerciasis (river blindness), transmitted by blackflies. Worldwide, an estimated 52 million people had lymphatic filariasis in 2018, and the disease predominantly occurs in the tropical countries of Africa and Asia [25]. Onchocerciasis, predominantly found in Africa and in foci in Yemen and Latin America, affects 21 million people, causing severe skin disease and vision loss [26]. The drugs diethylcarbamazine or ivermectin used to treat lymphatic filariasis and onchocerciasis mainly kill the larvae in the blood or skin but not the adult worms [26]. Because the nematodes are dependent on *Wolbachia* spp. for development and adult worm survival, DOX or rifampicin, which both deplete the endobacteria from the worms, can also be used, with the benefit that the targeting of *Wolbachia* spp. leads to the killing of adult worms and, therefore, does not require annual or biannual treatment for the lifetime of the adult worms as it is required for diethylcarbamazine and ivermectin [23,24,27]. Anti-wolbachial treatment is the only safe and effective adult worm-killing therapy [28], and new drugs without the contraindications for DOX and rifampicin are needed.

Treatment failure of chlamydial infections, the high prevalence of *C. trachomatis* and diseases caused by filarial nematodes, and long treatment regimens in combination with low compliance and contraindications for filarial infections stress the need for new antimicrobial treatment strategies against *Chlamydia* and *Wolbachia*. An important antibiotic target in many bacteria is the peptidoglycan (PGN) layer within the cell envelope. PGN is unique to bacteria and usually consists of linear glycan strands connected by cross-linked peptides that form an exoskeleton-like meshwork that protects bacteria from osmotic stress. Inside their intracellular niche, *Chlamydia* and *Wolbachia* are protected from osmotic challenges, and the reductive adaptation to their host is reflected by the loss of a canonical energy-cost-intensive PGN-based cell wall [29,30,31].

However, both endobacteria retained reduced, but functional, PGN biosynthesis pathways that have been implicated in cell division [32,33,34]. *Chlamydia* synthesize a transient PGN ring that is required for a unique cell division process [29,30,35,36]. The exact PGN structure in *Wolbachia* is unknown, but a growing body of research provides evidence for a reduced PGN-like structure that is also important for cell division [31,32,33].

Antibiotics targeting PGN biosynthesis in the obligate intracellular *Chlamydia* and *Wolbachia* need to traverse several membranes, and due to the reduced synthesis machinery and distinct functions and structure of PGN in both endobacteria, these antibiotics may not act in the same manner as it has been defined in free-living bacteria. PEN binds to penicillin-binding proteins (PBPs) and thereby interferes with the transpeptidation and remodeling of PGN, which is usually bactericidal in many bacteria via not fully understood cellular downstream effects. PEN acts bacteriostatically in *Chlamydia* and induces a reversible state of persistence by blocking cell division, which leads to the formation of enlarged ABs that persist within the host cell [14,37,38,39]. *Wolbachia*, on the other hand, are unaffected by PEN treatment [40] but are susceptible to fosfomycin [32], which lacks activity against *Chlamydia* due to intrinsic resistance [41]. Fosfomycin targets the first synthesis step of the PGN precursor lipid II [42]. We previously showed that fosfomycin treatment also induces the formation of enlarged *Wolbachia* cells [33], a phenotype similar to that seen for PEN treatment of *Chlamydia*.

A target of interest within the lipid II synthesis pathway is the transferase MraY, a member of the polyprenyl-phosphate *N*-acetyl hexosamine 1-phosphate transferase superfamily. Our previous work showed that both *Chlamydia* and *Wolbachia* harbor functionally conserved MraY and MurG homologs and are thus capable of synthesizing the PGN precursor lipid II [32]. At the inner membrane of these two organisms, the soluble precursor uridine diphosphate (UDP)-*N*-acetylmuramoyl(Mur*N*Ac)-pentapeptide is first linked to the membrane carrier undecaprenyl-phosphate (bactoprenol, C_55_-P) by the integral membrane protein MraY, yielding the penultimate PGN precursor, lipid I. Subsequently, the glycosylation of lipid I with UDP-*N*-acetylglucosamine (Glc*N*Ac) by MurG leads to the formation of lipid II [32].

MraY homologs from different free-living bacteria, e.g., *Bacillus subtilis*, *Staphylococcus aureus*, and *Aquifex aeolicus*, were shown to be inhibited by naturally occurring nucleoside antibiotics of *Streptomyces* [43,44,45,46] that are comprised of different subclasses. Two of these are muraymycins and caprazamycins, which both contain a 6′-*N*-alkyl-5′-beta-*O*-aminoribosyl-C-glycyluridine structure and display high activity against several Gram-positive pathogens, e.g., methicillin-resistant *S. aureus*, vancomycin-resistant *Enterococcus* strains, and *Pseudomonas aeruginosa* [45,47,48,49]. Nucleoside antibiotics such as muraymycin (MRY) D2 are competitive inhibitors for the natural substrate of MraY, UDP-Mur*N*Ac-pentapeptide [45,47,50], due to the structural similarity of UDP-Mur*N*Ac to the nucleotide moiety of muraymycins. In contrast, the peptidic moiety of MRY D2 is non-competitive. During inhibition, a conformational change of the active site enables the binding of MRY D2 to MraY from *Aquifex aeolicus* [50].

In this study, we aimed to understand the PGN synthesis machinery in *Chlamydia* and *Wolbachia* and to explore the antimicrobial activity of muraymycins against these distinct non-model bacteria that have both functionally conserved the target of nucleoside antibiotics. Using cell culture-based infection models, we showed that these endobacteria are susceptible to muraymycins. In both genera, the MraY inhibitors induced the formation of enlarged cells that are similar to the chlamydial PEN-induced persistence phenotype. In complementary enzymatic studies with purified chlamydial MraY, we verified the integral membrane protein as a target of muraymycin D2 in *Chlamydia*. Based on our in silico analysis, we propose a similar mechanism for the binding of MRY D2 and its derivatives to the target protein MraY in *Chlamydia* and *Wolbachia* as that described for *A. aeolicus* MraY. When applied to PEN-induced persistent chlamydial infection, MRY D2 had persistence-breaking properties and eradicated the enlarged non-dividing chlamydiae from the host cells, indicating that the nucleoside antibiotics may have further effects in addition to the inhibition of MraY.

## 2. Results

### 2.1. Muraymycin D2 and its Derivatives Induce the Formation of Enlarged Cells in C. trachomatis upon Treatment at an Early Stage of the Chlamydial Developmental Cycle

Treatment failure of genital chlamydial infections is not completely understood and needs to be addressed by understanding the underlying reasons and by searching for new antibiotics against the minimalist bacteria. Here, we explored the effects of muraymycins in obligate intracellular *Chlamydia*. These natural compounds block PGN synthesis through interference with the transferase MraY in free-living bacteria [45,47,48,49,50]. Since the enzyme is functionally conserved in *Chlamydia* [32], we postulated that muraymycins may have anti-chlamydial activity and hamper biosynthesis of the PGN ring that is essential in chlamydial cell division, as shown for the persistence-inducing antibiotic PEN.

**Figure 1:**
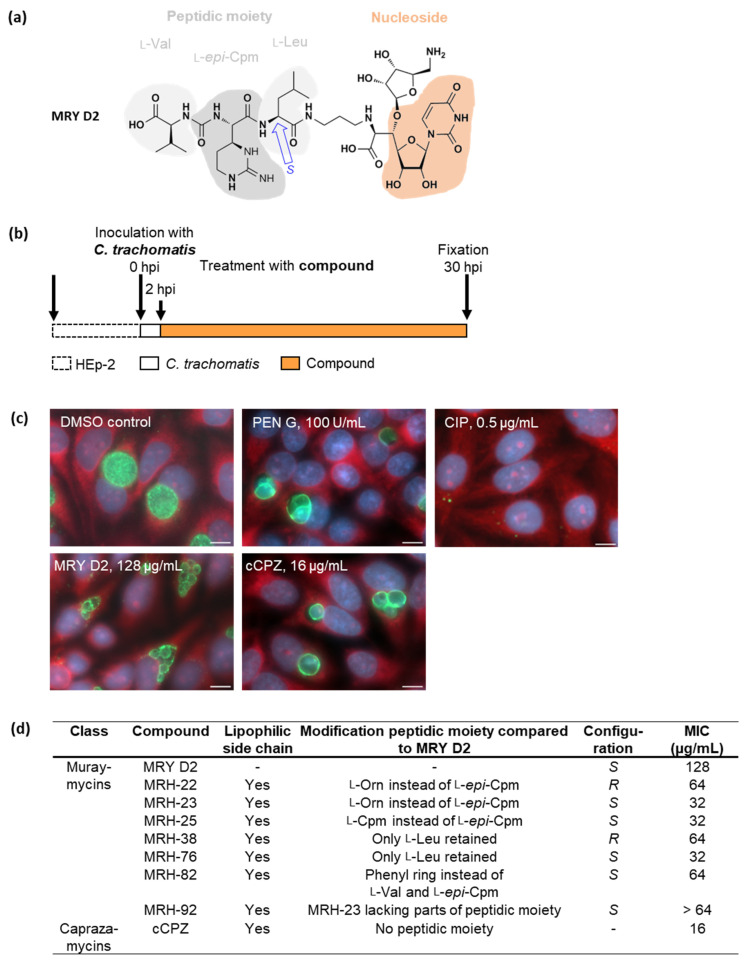
Effects of muraymycin (MRY) D2, its derivatives, and carbacaprazamycin (cCPZ) on *C. trachomatis*. (**a**) The lead compound MRY D2 consists of a nucleoside and peptidic moiety made up of l-Val, l-*epi*-capreomycidine (Cpm), and l-Leu. The bond between the latter two has an *S*-configuration. (**b**) HEp-2 cells were infected with *C. trachomatis* D/UW-3/CX, the compounds were added at 2 h post infection (hpi), and anti-chlamydial activity was analyzed at 30 hpi by fluorescence microscopy. (**c**) The vehicle control with dimethyl sulfoxide (DMSO) showed an active infection, 100 U/mL penicillin (PEN) G induced persistence visible as enlarged, aberrant bodies (ABs), while 0.5 µg/mL ciprofloxacin (CIP) was bactericidal. MRY D2 is shown at the minimal inhibitory concentration (MIC; 128 µg/mL), at which the formation of aberrant cells was induced; cCPZ had the lowest MIC of 16 µg/mL. Eukaryotic host cell cytoplasm from representative images was labeled with Evans Blue (red), genomic desoxyribonucleic acid (DNA) with 4′,6-diamidino-2-phenylindole (DAPI; blue), and chlamydial lipopolysaccharide with fluorescein (green). Representative images of three experiments. Scale bar: 10 µm. (**d**) Overview of the structural features of muraymycin derivatives (MRH) and cCPZ compared with MRY D2. MRH-22 and -23 have an l-ornithine (l-Orn). Compared with MRY D2, the MICs of muraymycin and caprazamycin derivatives were lower, ranging between 16 µg/mL and 64 µg/mL. Data obtained from three experiments. MRH-92 did not induce the formation of enlarged chlamydial cells.

First, we tested the lead compound MRY D2 (Figure 1a) and seven muraymycin derivatives (MRH; described in [47,48]) on *C. trachomatis* in cell culture experiments. To this end, *C. trachomatis* D/UW-3/CX-infected HEp-2 cells were treated with the compounds at 2 h post infection (hpi; Figure 1b), which represents an early stage of the chlamydial developmental cycle before bacterial replication [14,51]. Inhibitors were applied over a range of concentrations that were not detrimental to HEp-2 host cells in resazurin-based viability assays (Table S1), and the effects were visualized by fluorescence microscopy (Figure 1c and Figure S2) at 30 hpi. The minimal inhibitory concentration (MIC) for *Chlamydia* was defined as either the lowest concentration leading to a >90% reduction in inclusions [52] or, for aberrance-inducing compounds, the concentration leading to abnormal chlamydial cell phenotypes, i.e., enlarged cells after treatment with PEN G [39].

As expected, our data showed that the PEN G control acted bacteriostatically, resulting in the presence of enlarged, non-dividing bacterial cells in the inclusion, whereas the ciprofloxacin control killed the pathogens and cleared the infection from the host cells (Figure 1c). The lead compound, MRY D2 (Figure 1a), induced the formation of large ABs at 128 µg/mL (Figure 1c), phenocopying the PEN-induced persistence phenotype (Figure 1c). This observation supports our hypothesis that MRY D2 targets chlamydial MraY and thereby affects the cell division machinery in the cell-wall-lacking minimalist endobacterium.

Compared with MRY D2, all muraymycin derivatives had a lipophilic C_15_ side chain (Figure 1d and Figure S1) added to their peptidic moiety. Each derivative had further modifications in the peptidic moiety, and some were configuration isomers (MRH-22/23; MRH-38/-76; Figure 1d and Figure S1). Except for MRH-92, treatment with all other muraymycin derivatives led to an enlarged chlamydial cell phenotype (Figure S2). However, the phenotype was induced at lower concentrations compared with the lead compound: MRH-22, -38, and -82 had an MIC of 64 µg/mL, while MRH-23, -25, and -76 had an MIC of 32 µg/mL (Figure 1d). Since these derivatives induced the phenotype at lower concentrations, the addition of the lipophilic side chain seemed to enhance the anti-chlamydial activity. Furthermore, the anti-chlamydial activity was increased two-fold for the derivatives MRH-23 and -76, which had an *S*- instead of an *R*-configuration in the bond linking the acylated l-leucine to the peptidic moiety.

The derivative MRH-92 did not induce the formation of aberrant chlamydial cells even at the highest tested concentration of 64 µg/mL (Figure S2). The phenotype of the inclusions resembled that of the dimethyl sulfoxide (DMSO) vehicle control (Figure 1c). We could not explore whether higher concentrations of MRH-92 might have led to the abnormal phenotype observed for the other muraymycins due to host cell cytotoxic effects at concentrations > 64 µg/mL (Table S1).

In addition, we analyzed the effect of another nucleoside antibiotic on *C. trachomatis*, the caprazamycin-derivative carbacaprazamycin (cCPZ; described in [49]) (Figure S1). Like most of the tested muraymycins, cCPZ induced the formation of enlarged, aberrant chlamydial cells with an MIC of 16 µg/mL, the lowest MIC measured in this study (Figure 1c,d).

Taken together, the majority of muraymycins and cCPZ induced the formation of enlarged *C. trachomatis* cells. In free-living bacteria, these antibiotics inhibit MraY and arrest cell wall biosynthesis [45,47,48,49,50]. Based on our cell culture data obtained with *C. trachomatis*, we propose that the natural compounds also block cell division in this cell-wall-lacking non-model bacterium through interference with transferase MraY.

### 2.2. Muraymycins Induce the Formation of Enlarged Cells in Wolbachia

*Chlamydia* and *Wolbachia* are both intracellular bacteria with reduced genomes and modified PGN synthesis and cell division machineries. We showed that muraymycins induce the formation of enlarged cells in *C. trachomatis*; thus, we wanted to analyze if there are similar effects on the endosymbiont *Wolbachia* of *Aedes albopictus* (mosquito) cells. First, we assessed the cytotoxicity of the compounds toward the insect cells by quantitative real-time polymerase chain reaction (qPCR) to detect the insect *actin B* gene. Infected *A. albopictus* insect cells were cultured with varying concentrations of the derivatives or with the protein biosynthesis inhibitor DOX as a positive control for nine days. Most of the compounds were detrimental to the host cells; however, MRH-76 and -92 were non-toxic at the tested concentration range.

**Figure 2:**
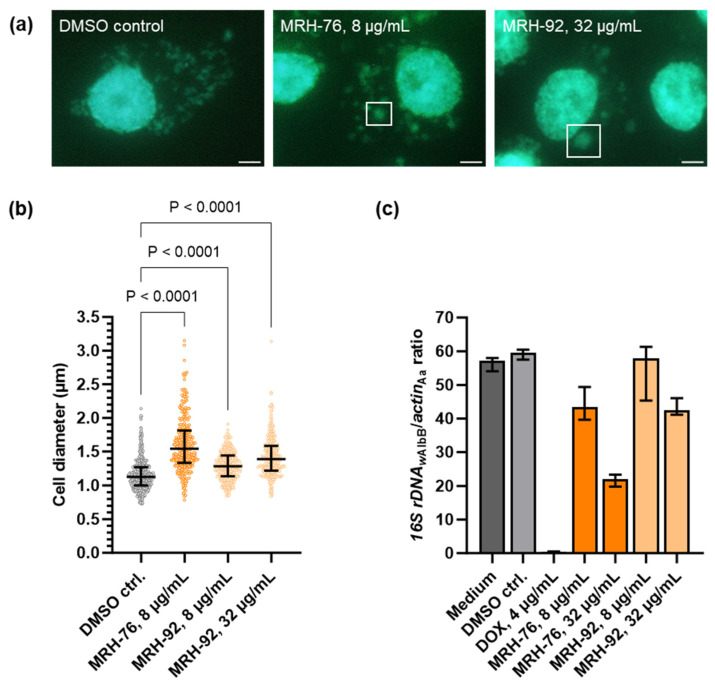
Effects of the muraymycin derivatives MRH-76 and -92 on *Wolbachia* strain B of *Aedes albopictus*. (**a**) *A. albopictus* C6/36 cells infected with *Wolbachia* strain B of *A. albopictus* were cultured for 9 days with or without the compounds at varying concentrations. Treatment with 8 µg/mL MRH-76 or 32 µg/mL MRH-92 induced the formation of enlarged *Wolbachia* cells (exemplary cells indicated by white squares). Genomic DNA was stained with DAPI (green). Scale bar = 2 µm. (**b**) Using ZEN 3.5 (blue edition) with the image analysis module, the diameter of untreated and treated *Wolbachia* from 3 to 4 images was determined, shown as median ± interquartile ranges. For statistical analysis, a Kruskal–Wallis test was applied; *p* < 0.0001. *n* = 663 (control), 294 (MRH-76, 8 µg/mL), 348 (MRH-92, 8 µg/mL), and 298 (MRH-92, 32 µg/mL) cells. The cell diameter of *Wolbachia* cells treated with 8 µg/mL MRH-76, as well as of those treated with 8 µg/mL and 32 µg/mL MRH-92, was significantly larger compared with the vehicle control. (**c**) The depletion of *Wolbachia* from infected and treated C6/36 cells was measured after 9 days by extracting genomic DNA and measuring the ratio of *Wolbachia 16S rDNA* and *A. albopictus actin B* by qPCR; the median ± interquartile range from three experiments is shown. *Wolbachia* were nearly completely depleted by 4 µg/mL doxycycline (DOX), the positive control, whereas treatment with MRH-76 and MRH-92 led to a concentration-dependent reduction of *Wolbachia*, with 32 µg/mL MRH-76 showing a >50% reduction compared with the negative DMSO control.

Therefore, we analyzed the activity of these two muraymycins using fluorescence microscopy and by measuring bacterial depletion by qPCR to detect the *16S rDNA* gene from *Wolbachia*, normalized to the *actin B* gene from *A. albopictus*. As *Wolbachia* grow more slowly than *Chlamydia*, a longer treatment time was needed to see potential effects of the inhibitors [53]. Our microscopy data showed that in untreated and DMSO-treated insect cells, *Wolbachia* were distributed throughout the host cell cytoplasm, seen as small green fluorescent foci (Figure 2a). Whilst treatment with 4 µg/mL DOX led to a depletion of the bacteria from the host cells (Figure 2c), treatment with 8 µg/mL MRH-76 led to the formation of enlarged wolbachial cells (Figure 2a; exemplary cells indicated by white squares), similar to the fosfomycin-induced *Wolbachia* phenotype [33]. Interestingly, enlarged *Wolbachia* also appeared upon treatment with 32 µg/mL MRH-92 (Figure 2a), which had no effect on *C. trachomatis* (Figure 1c).

The diameter of *Wolbachia* cells was determined from the microscopy images, and a significant increase was found for 8 µg/mL MRH-76 as well as for 8 µg/mL and 32 µg/mL MRH-92 compared with the DMSO control (Figure 2b). While control cells had a median diameter of 1.13 µm, cells treated with 8 µg/mL MRH-76 were 37% larger (1.54 µm), and those treated with 8 µg/mL and 32 µg/mL MRH-92 were 13% (1.28 µm) and 23% (1.39 µm) larger, respectively. The proportion of enlarged cells was 24% for the control, 82% for 8 µg/mL MRH-76, and 53% and 65% for 8 µg/mL and 32 µg/mL MRH-92, respectively.

The relative bacterial quantity was measured by qPCR and revealed that *Wolbachia* were reduced in a concentration-dependent manner compared with the DMSO control. (Figure 2c). While treatment with 8 µg/mL MRH-92 did not affect the abundance of the bacteria, 32 µg/mL led to a 24% reduction. The same decrease was seen for cells treated with 8 µg/mL MRH-76, whereas 32 µg/mL MRH-76 reduced the intracellular organisms by 68% (Figure 2c). Compared with doxycycline, the muraymycin derivatives were less potent, requiring concentrations higher than 4 µg/mL to achieve depletion of *Wolbachia* over the same treatment time (Figure 2c).

As the treatment of *Wolbachia* with muraymycin derivatives had similar effects to fosfomycin, which targets the first enzyme of UDP-Mur*N*Ac-pentapeptide synthesis, our results support MraY as the target of muraymycins in *Wolbachia*.

### 2.3. Muraymycin D2 and the Derivative MRH-92 Inhibit the Chlamydial Transferase MraY In Vitro

**Figure 3:**
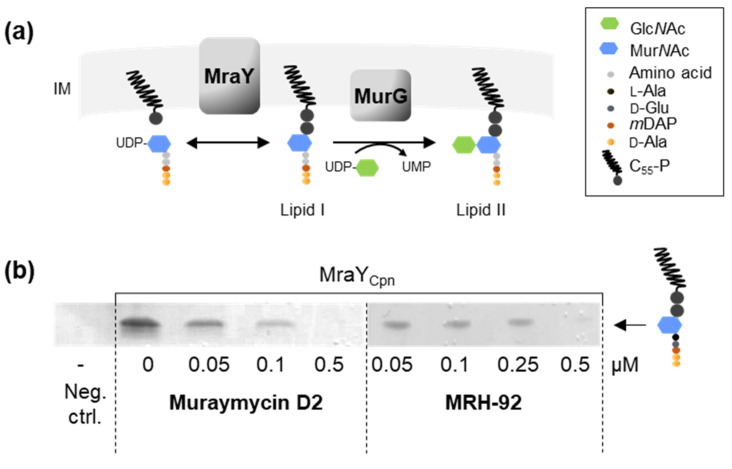
Formation of lipid I by the phospho-Mur*N*Ac-pentapeptide transferase MraY is inhibited by the muraymycins MRY D2 and MRH-92. (**a**) *C. pneumoniae* and *Wolbachia* spp. MraY use the precursor molecules undecaprenyl-phosphate (C_55_-P) and uridine diphosphate (UDP)-*N*-acetylmuramoyl(Mur*N*Ac)-pentapeptide to form lipid I, which is glycosylated with *N*-acetylglucosamine (Glc*N*Ac) by MurG, yielding lipid II. IM: inner membrane; UMP: uridine monophosphate; mDAP: *meso*-diaminopimelic acid; C_55_-P: undecaprenyl-phosphate. (**b**) Recombinant MraY_Cpn_ was incubated with C_55_-P, UDP-Mur*N*Ac-pentapeptide, and different concentrations of either MRY D2 or MRH-92 for 90 min at 37 °C. Reaction products were extracted, separated via thin-layer chromatography, and stained. Formation of lipid I by MraY_Cpn_ was inhibited by both tested inhibitors. MRY D2 showed a concentration-dependent inhibition, with MraY_Cpn_ being completely inhibited at 0.5 µM. MRH-92 also inhibited the enzymatic reaction at 0.5 µM.

As shown above, muraymycins induced the formation of enlarged cells in both intracellular species *C. trachomatis* and *Wolbachia*. Therefore, we hypothesized that MRY D2 and the tested derivatives target the transferase MraY (Figure 3a) in *Wolbachia* and *Chlamydia* as in free-living bacteria [45,47,48,49,50,54]. We previously showed that recombinant purified MraY of *C. pneumoniae* and *Wolbachia* endosymbiont from *B. malayi* exhibit activity in vitro, leading to the formation of lipid I using bactoprenol and UDP-Mur*N*Ac-pentapeptide [32]. Inhibition of MraY by compounds such as MRY D2 would lead to a depletion of lipid I and thereby block the synthesis of new PGN material. As a result, cell division would be arrested, similar to that seen following treatment with other antibiotics such as PEN G and fosfomycin in *Chlamydia* and *Wolbachia*, respectively [14,33]. To further support the hypothesis that inhibition of MraY by MRY D2 and its derivatives caused the formation of enlarged cells, we performed MraY enzyme inhibition assays.

*C. pneumoniae* MraY was heterologously overexpressed in *E. coli*, purified via His-tag affinity chromatography, and inhibition of the enzymatic activity was analyzed by visualizing the reaction products via thin-layer chromatography. We conducted MraY_Cpn_ inhibition assays by incubation with either MRY D2 or MRH-92 at concentrations between 0.05 µM and 0.5 µM, as the compounds did not need to traverse four membranes as in the cell culture-based experiments, and lower concentrations could be used. Both compounds completely abolished enzymatic activity at 0.5 µM (Figure 3b), indicating that MraY_Cpn_ is the target of MRY D2 and MRH-92.

### 2.4. In Silico Analysis Reveals That Chlamydia and Wolbachia MraY Retain All Amino Acid Residues Important for Binding of UDP-MurNAc-Pentapeptide and Muraymycins

To further support the hypothesis that MraY of *Chlamydia* and *Wolbachia* is inhibited by muraymycins, we performed in silico studies. These included protein sequence alignments and 3D-protein model predictions with phyre2 created for MraY of *C. trachomatis* (Ctr), *C. pneumoniae* (Cpn), and *Wolbachia* endosymbionts of *A. albopictus* (*w*AlbB) and *B. malayi* (*w*Bm) against crystal structures of *A. aeolicus* (Aae) MraY. The MraY_Aae_ active site has been well studied [55,56,57,58] in the apo form where the cofactor Mn^2+^ was exchanged for Mg^2+^ [58], as well as in complex with MRY D2 [50]. Additionally, crystallization with cCPZ led to further characterization of residues contributing to the binding of the lipophilic side chain [54].

**Figure 4:**
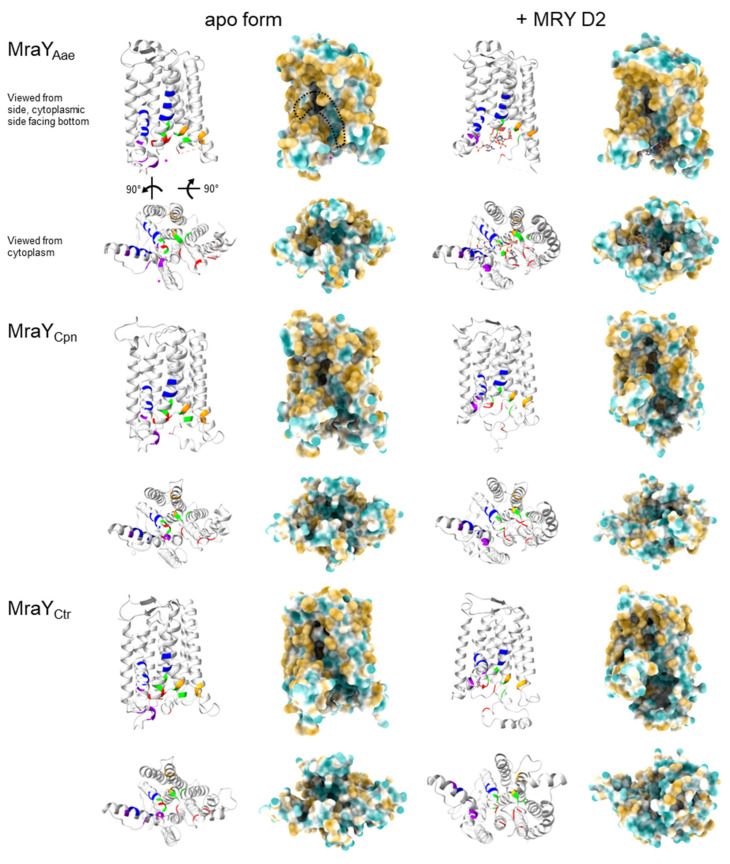

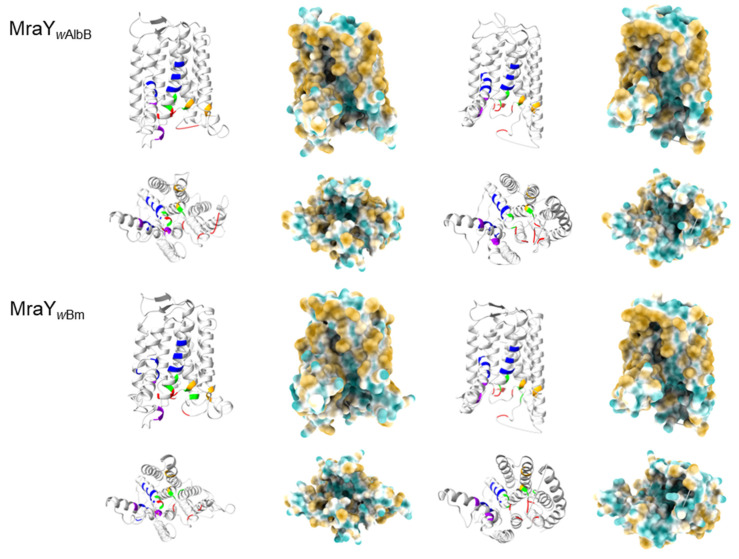
Structural comparison of *A. aeolicus* apoMraY and MraY bound to MRY D2, and predictions of *C. pneumoniae*, *C. trachomatis*, and *Wolbachia* endosymbionts of *A. albopictus* and *B. malayi* MraY and MraY bound to MRY D2. Bacterial strains and primary protein accession numbers: *A. aeolicus* VF5 (Aae, O66465), *C. trachomatis* D/UW-3/CX (Ctr, O84762), *C. pneumoniae* GiD (Cpn, A0A0F7WR30) and *Wolbachia* endosymbiont of *A. albopictus* (*w*AlbB, A0A4S2QUK2) and *B. malayi* (*w*Bm, Q5GRZ3). MraY structure predictions were made by pyhre2 against apoMrY_Aae_ chain A [58] or MraY_Aae_ crystallized with MRY D2 [50] with 100% confidence. Proteins are viewed from the side (top: periplasm; bottom: cytoplasm) or turned by 90° + 90° and viewed from the cytoplasm. Amino acid residues of the active site (orange), Mn^2+^ ions (representative for Mg^2+^; grey), Ni^2+^ ions (magenta), as well as residues contributing to binding of uracil (red), 5′-aminoribose (green), and the peptidic side chain (violet) from MRY D2 and the acyl moiety of cCPZ (blue), are highlighted as in [50,58]. Surface hydrophobicity is indicated from hydrophobic (brown) to hydrophilic (turquoise). The hydrophobic groove (dashed line) of apoMraY_Aae_ is visualized as in [58]. The phyre2 predictions revealed that MraY_Ctr/Cpn/*w*AlbB/*w*Bm_ have similar secondary and tertiary structures compared to apoMraY_Aae_. The active sites face the cytoplasm. In MraY_Ctr/Cpn/*w*AlbB/*w*Bm_, the uracil, 5′-aminoribose, and peptidic side chains face the cytoplasm, residues contributing to the binding of the cCPZ acyl chain are located at the surface of the protein groove as in apoMraY_Aae_. Residues V266 and I267 of MraY*_w_*_AlbB_, contributing to acyl moiety binding in MraY_Aae_, could not be predicted.

**Figure 5:**
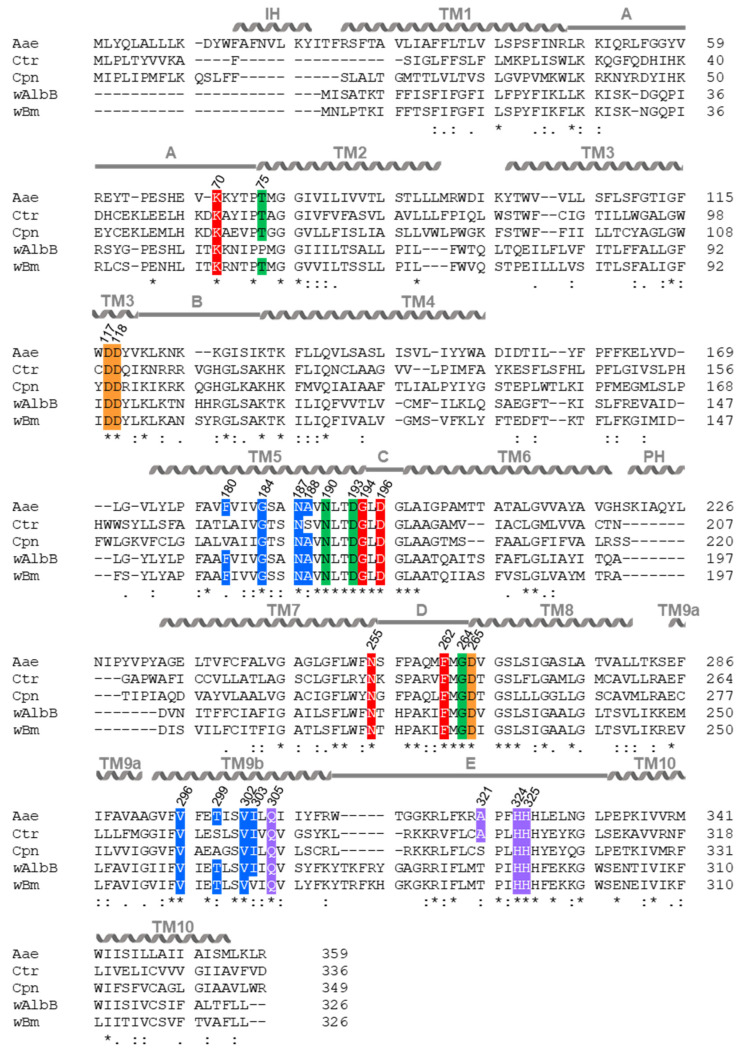
Sequence alignment of MraY from *A. aeolicus*, *C. trachomatis*, *C. pneumoniae*, and *Wolbachia*. Bacterial strains and primary protein accession numbers: *A. aeolicus* VF5 (Aae, O66465), *C. trachomatis* D/UW-3/CX (Ctr, O84762), *C. pneumoniae* GiD (Cpn, A0A0F7WR30) and *Wolbachia* endosymbiont of *A. albopictus* (*w*AlbB, A0A4S2QUK2) and *B. malayi* (*w*Bm, Q5GRZ3). Alignments were performed with Clustal Omega, the position of specific residues is indicated for MraY_Aae_. Compared with MraY_Aae_ [56,57,58], the three proposed catalytical aspartic acid residues (orange) and histidine (purple) residues within the active site of MraY_Ctr/Cpn/*w*AlbB/*w*Bm_ are highly conserved. MraY_Aae_ residues contributing to the binding of MRY D2 are indicated based on the respective MRY D2 moieties, which are uracil (red), 5′-aminoribose (green), and the peptidic side chain (violet) [50,54]. The acyl moiety of cCPZ is bound in a hydrophobic groove in MraY_Aae_ (blue) [54]. With a few exceptions, these residues are also conserved in MraY_Ctr/Cpn/*w*AlbB/*w*Bm_. MraY_Aae_ contains an interfacial helix (IH), ten transmembrane domains (TM), one periplasmic helix (PH), and five cytoplasmatic loops (A–E). Conservation grade: fully conserved (*), strongly similar properties (:), weakly similar properties (.).

Our analysis revealed that the MraY orthologs of *Chlamydia* and *Wolbachia* have varying similarities compared to MraY_Aae_. MraY of *w*AlbB and *w*Bm have the highest protein sequence identities with 41% and 40%, respectively, followed by MraY_Cpn_ with 37% and MraY_Ctr_ with 33%, respectively (Table S2). As for MraY_Aae_ [58], our protein models predicted ten transmembrane domains (TM) for the transmembrane enzymes MraY_Ctr/Cpn/*w*AlbB/*w*Bm_ (Figure 4 and Figure 5). The active sites are predicted to be located at the outside of the proteins, facing the cytoplasm, and are comprised of TM3, TM8, and the cytoplasmic loop E (Figure 4 and Figure 5), as shown for apoMraY_Aae_ [58]. In MraY_Aae_, the four essential catalytic residues are D117, D118, D265, and H324 (Figure 4 and Figure 5; indicated in orange) [56,57,58]. Several analyses with MraY_Aae_ indicated that D117 is involved in binding the phosphate moiety of the substrate C_55_-P [57,58], and D265 was shown to be involved in coordinating Mg^2+^ in apoMraY_Aae_ [58]. Our alignment of MraY_Ctr/Cpn/*w*AlbB/*w*Bm_ with MraY_Aae_ revealed that all four essential catalytic residues are highly conserved in *Chlamydia* and *Wolbachia*: residues D100, D101, D243, and H301 in MraY_Ctr_; D110, D111, D256, and H314 in MraY_Cpn_; and D94, D95, D229, and H293 in MraY*_w_*_AlbB/*w*Bm_ (Figure 5).

Crystal structures of MraY_Aae_ bound to MRY D2 showed that MRY D2 did not interact with the three catalytic aspartic acid residues of the active site but, rather, formed a water-mediated hydrogen-bonding network with H324 and H325, occupying the binding site for UDP-Mur*N*Ac-pentapeptide [50]. Upon MRY D2 binding, a conformational change of MraY_Aae_ took place, where the active site was reshaped forming a nucleoside-binding and a peptide-binding pocket, which still faced the cytoplasm (Figure 4) [50,58]. The lipophilic side chain of cCPZ was bound in a hydrophilic cleft (Figure 4) [54,58]. Thus, the mode of inhibition of MraY_Aae_ by MRY D2 and acylated derivatives was competitive for the natural substrate UDP-Mur*N*Ac-pentapeptide regarding the nucleotide-binding site but non-competitive for C_55_-P [47,50,54].

As in MraY_Aae_, our predictions for MraY_Ctr/Cpn/*w*AlbB/*w*Bm_ identified that the residues contributing to the binding of MRY D2 and the lipophilic side chain are located on the outside of the proteins and face the cytoplasm and hydrophobic cleft, respectively (Figure 4). Furthermore, MraY_Ctr/Cpn/*w*AlbB/*w*Bm_ seem to change their conformation as the active sites are wider in our respective predictions, leading to the formation of uracil and peptidic side-chain binding pockets by moving the residues for binding closer together (Figure 4), as shown for MraY_Aae_ [50].

Our models for MraY_Ctr/Cpn/*w*AlbB/*w*Bm_ further showed that the majority of residues contributing to the binding of uracil, 5′-aminoribose, and the peptidic moiety of MRY D2, as well as the binding of a lipophilic side chain, are also highly conserved and are located at the surface of the protein (Figure 4 and Figure 5; indicated in red, green, violet, and blue, respectively). In MraY_Ctr_, the amino acids F180, A188, and T299 of the lipophilic binding site of MraY_Aae_ are not conserved; in MraY_Cpn_, the residues F180 and T299 as well as A321 of the peptidic binding site of MraY_Aae_ are not present. The latter is also lacking in MraY*_w_*_AlbB/*w*Bm_. Additionally, in MraY*_w_*_AlbB_, T75 of the 5′-aminoribose binding site and in MraY*_w_*_Bm_, I303 of the lipophilic binding site of MraY_Aae_ are not conserved (Figure 4 and Figure 5).

Together, the high level of conservation at the primary, secondary, and tertiary structural levels gives further evidence for similar enzymatic and inhibitory mechanisms of muraymycins versus MraY_Ctr/Cpn/*w*AlbB/*w*Bm_ compared with MraY_Aae_. It is likely that MRY D2 and its derivatives fit into the binding pockets of the enzymes and thereby compete with the substrate UDP-Mur*N*Ac-pentapeptide, leading to inhibition of the enzyme and, thus, depletion of lipid I within the cell.

### 2.5. Muraymycin D2 Eliminates Persistent C. trachomatis Cells from an Established PEN-Induced Persistent Infection upon Addition at the Mid-Stage of the Chlamydial Developmental Cycle

As we have shown, muraymycins target MraY and interfere with the cell division process in both *Chlamydia* and *Wolbachia*, leading to enlarged cells, phenocopying the PEN-induced persistent infection in *Chlamydia* [14] and the fosfomycin-induced phenotype in *Wolbachia* [33]. The mechanism behind the PEN-induced persistence in *Chlamydia* is not entirely understood. Because muraymycins and PEN G have different structural targets in PGN synthesis, we investigated the effects of simultaneous treatment with MRY D2 and PEN G on a chlamydial infection and if MRY D2 has an effect on an established PEN G-induced persistent *C. trachomatis* infection. The latter would also be of interest in the context of the treatment of chlamydial infections in vivo, where enlarged chlamydial cells have been observed and treatment failures with first-choice antibiotics have been reported [6,12,16,17,18].

**Figure 6:**
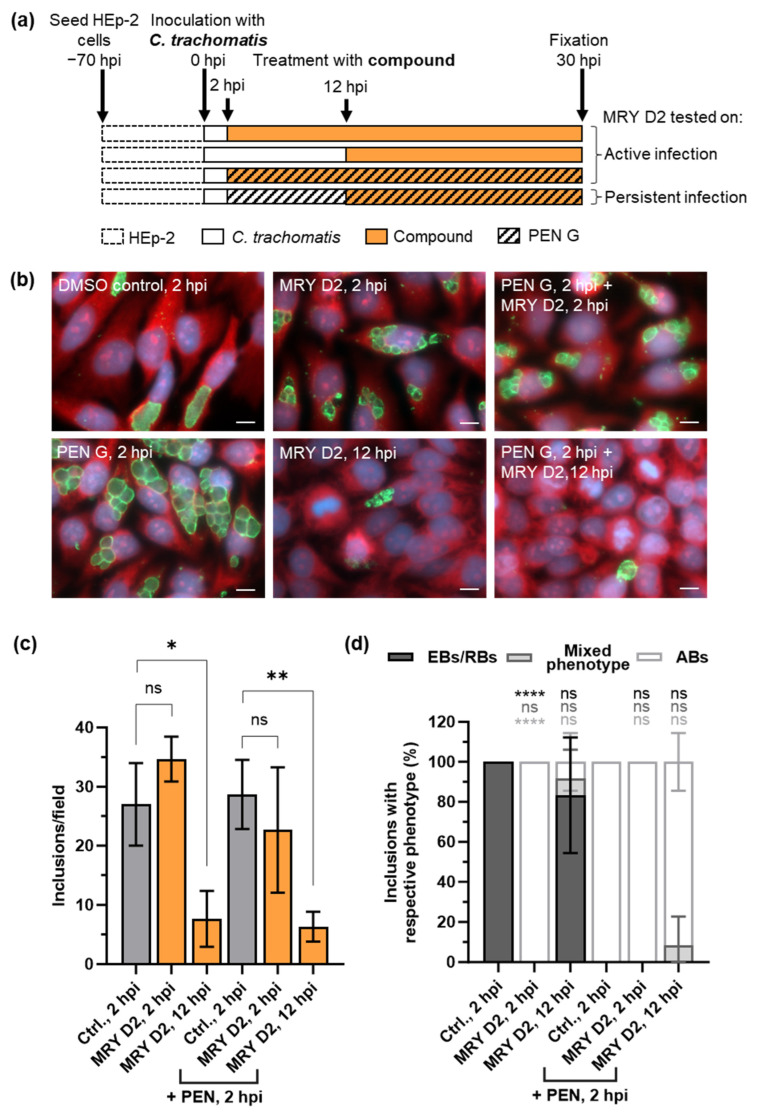
Effect of MRY D2 on PEN G-induced persistent *C. trachomatis* infections. (**a**) HEp-2 cells infected with *C. trachomatis* D/UW-3/CX were treated with 128 µg/mL MRY D2 at 2 hpi or 12 hpi to analyze the effect on active infection. Additionally, 128 µg/mL MRY D2 and 100 U/mL PEN G were added simultaneously at 2 hpi. To assess the effect on a PEN G-induced persistent infection, 100 U/mL PEN G was added at 2 hpi, and 128 µg/mL MRY D2 was added at 12 hpi. Anti-chlamydial activity was analyzed at 30 hpi by fluorescence microscopy. (**b**) Eukaryotic host cell cytoplasm from representative images was labelled with Evans Blue (red), genomic DNA with DAPI (blue), and chlamydial lipopolysaccharide with fluorescein (green). Scale bar: 10 µm. (**c**) Mean ± SD of inclusions/field and (**d**) the relative number of inclusions filled with either elementary bodies (EBs)/reticulate bodies (RBs), ABs, or a mixed phenotype, analyzed from three images. An unpaired, two-tailed Student’s *t*-test was performed against the respective vehicle control. ns: not significant, *p* > 0.05; *: *p* = 0.05 to 0.01; **: *p* = 0.01 to 0.001; ****: *p* < 0.0001. In comparison with the DMSO vehicle control, the addition of PEN G at 2 hpi induced persistence, characterized by aberrant bodies. Early application of MRY D2 at 2 hpi (*p* < 0.0001) and simultaneous application with PEN G at 2 hpi induced the formation of ABs. Application of MRY D2 at mid-phase (12 hpi) did not lead to the formation of ABs, but fewer inclusions were observed (*p* = 0.0166). This was also visible if persistence was induced by PEN G at 2 hpi followed by MRY D2 addition at 12 hpi (*p* = 0.0037).

To study simultaneous treatment, we added 100 U/mL PEN G and 128 µg/mL of MRY D2 at 2 hpi to *C. trachomatis* D/UW-3/CX-infected cells to analyze any effects of a combined administration (Figure 6a). These experiments revealed that when MRY D2 and PEN G were added simultaneously, inclusions filled with ABs were visible as for PEN G alone (Figure 6b–d).

We next investigated the effects of MRY D2 on an established persistent infection by inducing persistence with PEN G at 2 hpi followed by the addition of MRY D2 at 12 hpi (Figure 6a). At this time point, chlamydial cells are usually in the mid-phase of their developmental cycle and have started replicating [14,51]. In cells treated with PEN G at the beginning of the infection, ABs are visible after 12 h [14]. After the application of MRY D2 at 12 hpi to the PEN G-induced persistent *C. trachomatis* infection, fewer inclusions containing ABs were observed (Figure 6b,d), corresponding to a 70% reduction of the inclusion quantity (Figure 6c). As a control, MRY D2 was also added alone at 12 hpi (Figure 6a) to the active infection at the mid-stage of the chlamydial developmental cycle. Again, 75% fewer inclusions were observed, of which 83% contained EBs/RBs and 8% contained a mixed phenotype (EBs/RBs/ABs) (Figure 6b,c).

In summary, we saw that the mid-stage application of MRY D2 to dividing or persisting *Chlamydia* results in clearance of the inclusions by the host cell.

## 3. Discussion

The endobacteria *Chlamydia* spp. and *Wolbachia* spp. are of medical importance. Depending on the serovar, *C. trachomatis* can cause eye infections and infections of the ano-urogenital tract. Chronic or untreated infections can have detrimental long-term effects, e.g., blindness (serovar A-C) [1] or infertility in women (serovar D-K) [3,4]. Genital chlamydial infections are treated with DOX or AZI [5]; however, an 8% treatment failure rate is reported [8,9], which might occur due to re-infection from the gastro-intestinal tract [10] or persistent *C. trachomatis* cells [6,12]. Persisting chlamydial cells were shown to be less susceptible to DOX and AZI in murine models and cell culture experiments [11,16,17,18]. *Wolbachia* reside in the filarial nematodes *W. bancrofti*, *B. malayi*, *B. timori*, and *O. volvulus* [19,20]. These nematodes cause the neglected tropical diseases lymphatic filariasis (lymphedema or hydrocele) and onchocerciasis (severe skin disease or vision loss). Anti-wolbachial treatment with DOX or rifampicin is the only effective and safe treatment against adult worms as *Wolbachia* are obligate endosymbionts that support filarial development, growth, and survival [23,24,27]. Overall, contraindications for these antibiotics, including long treatment regimens for the anti-wolbachial treatment of filariasis, and treatment failure in *Chlamydia* require new strategies to fight these infections.

In this study, we explored the activity of the nucleoside antibiotics muraymycins and caprazamycins against *Chlamydia* and *Wolbachia*. Muraymycins and caprazamycins share a core structure comprised of glycyluridine, to which an aminoribose is attached at the 5′ position [43,44,46]. In MRY D2, an *N*-alkylamide linker is attached to the 6′ position of the nucleoside and connects the core structure with a peptidic moiety consisting of l-Leu, l-*epi*-capreomycidine, and l-Val [43]. Carbacaprazamycins, which have an acyl chain linked directly to the diazepanone ring, are chemically more stable derivatives of caprazamycin [49]. Nucleoside antibiotics display structural similarity to UDP-Mur*N*Ac-pentapeptide, the natural substrate of the transferase MraY, which catalyzes the formation of lipid I. This is the penultimate precursor molecule of bacterial PGN. Due to the structural similarity of nucleoside antibiotics and UDP-Mur*N*Ac-pentapeptide, the antibiotics are competitive for the MraY binding site of the substrate [47,50,54] and thereby inhibit enzymatic activity, as shown for multiple species, e.g., *B. subtilis* and *S. aureus* [45,48,49]. Binding of nucleoside antibiotics to MraY blocks lipid I formation and halts bacterial PGN synthesis.

Inside their intracellular niches, *Chlamydia* and *Wolbachia* are protected against osmotic stress [32], making a canonical cell wall redundant. However, both genera have retained all genes for *de novo* synthesis of the ultimate PGN building block, lipid II [32,33]. We previously hypothesized that lipid II synthesis is essential for cell division of *Chlamydia* and *Wolbachia* and demonstrated functional conservation of the transferase MraY and the transglycosylase MurG, which catalyze the formation of lipid I and lipid II, respectively [32]. Furthermore, some enzymes involved in the assembly, modification, and recycling of PGN are conserved [33,34]. Later, it was shown that *Chlamydia* synthesizes a transient PGN ring that facilitates proper cell division initiated by a specialized budding mechanism [29,30,35,36], whereas the exact PGN structure in *Wolbachia* is still unknown. However, research in this field proposes a reduced PGN-like structure [31,32,33]. Based on these observations, we postulated that nucleoside antibiotics, including muraymycins, exhibit antibacterial activity against the minimalist endobacteria *Chlamydia* and *Wolbachia* via inhibition of MraY, leading to interference of the PGN synthesis and cell division machineries. 

To investigate the effects of the nucleoside antibiotics against *C. trachomatis*, we tested MRY D2, seven muraymycin derivatives, and cCPZ, a caprazamycin derivative [47,48,49]. In contrast to MRY D2 (Figure 1a), all derivatives possessed a lipophilic C_15_ side chain (Figure 1d and Figure S1). Furthermore, the l-*epi*-capreomycidine in the peptidic moiety of MRY D2 was exchanged for l-capreomycidine in MRH-25 and for l-ornithine in MRH-22 and -23, respectively. In MRH-38 and -76, the whole peptidic moiety was deleted, while in MRH-82 and MRH-92, the peptidic moiety was reduced to a phenyl group and an l-ornithine, respectively, (Figure 1d and Figure S1). The compounds MRH-23/MRH-25 and MRH-38/MRH-76 are configuration isomers. Of note, all compounds retained the uracil, 5′-aminoribose, and 3′ hydroxyl group of the 5’ aminoribose, which are essential for activity, as shown for caprazamycins and lipsidomycins, another class of nucleoside antibiotics [59,60,61].

The tested compounds were applied to *C. trachomatis*-infected cells at an early stage of the chlamydial developmental cycle (Figure 1a), before the bacteria started replicating [14,51]. During the early treatment with MRY D2, the majority of muraymycin derivatives and cCPZ had MICs between 16 µg/mL for cCPZ and 128 µg/mL for MRY D2 (Figure 1d). These MICs were relatively high compared with the MICs of the first-choice antibiotics against uncomplicated chlamydial genital infections, DOX and AZI, which have MICs of <1 µg/mL against *C. trachomatis* serovar D in cell culture [62,63]. All derivatives harboring the lipophilic side chain had more potent anti-chlamydial activity compared with MRY D2 as reflected by their lower MICs (Figure 1d). This is probably due to the increased membrane permeability of the derivatives, as the compounds have to pass four membranes to reach their target MraY in *Chlamydia*. The same effect was also described for free-living pathogens [47].

The stereochemistry of the bond linking the peptidic moiety to the nucleoside core structure (Figure 1d and Figure S1) influenced the activity of the compound, as described in *S. aureus*, *Enterococcus faecalis*, and *Enterococcus faecium* [45,47]. If the bond had an *S*-configuration, as in the isomers MRH-23 and -76 (32 µg/mL), the MIC for *Chlamydia* was two-fold lower compared with the *R*-configuration in the isomers MRH-22 and -38 (64 µg/mL, Figure 1d). Another structural feature that influenced the activity in *S. aureus*, *E. faecalis*, and *E. faecium* were the amino acids of the peptidic moiety. The activity was two- to four-fold higher when the peptidic moiety included l-capreomycidine instead of l-ornithine [47]. This seemed to be less relevant in *C. trachomatis*, as reflected by the same MIC for MRH-25 and -23 (64 µg/mL), which had the same structure but contained l-capreomycidine and l-ornithine, respectively (Figure 1d and Figure S1). Furthermore, other studies revealed the importance of l-leucine of the peptidic moiety for the activity of the derivatives [45,47]. Exchanging the accessory peptidic motif, including l-leucine and the *N*-alkylamide linker, for a C_12_ side chain led to a much weaker activity similar to MRY D2 in *S. aureus*, *E. faecalis*, and *E. faecium* [45,47]. In our study, the muraymycin derivatives with the most reduced structure were MRH-38 and -76, which only retained the acylated l-leucine (Figure 1d and Figure S1). They exhibited similar anti-chlamydial activity (64 µg/mL and 32 µg/mL) as the other derivatives (Figure 1d), highlighting the importance of the peptidic moiety for interacting with the target enzyme and thereby increasing the antibacterial activity of these derivatives. Based on our observations, improved anti-chlamydial activity of a muraymycin is obtained by acylation, which increases the membrane permeability, and by having an *S*-configuration of the bond linking l-leucine to the rest of the peptidic moiety. The lowest MIC measured for *C. trachomatis* was for cCPZ (Figure 1d). As proposed by others, the diazepanone ring might contribute to the higher anti-chlamydial activity by positioning the uridine, 5′-aminoribose, and acyl chain to the respective binding sites in MraY [49,54,59].

**Figure 7:**
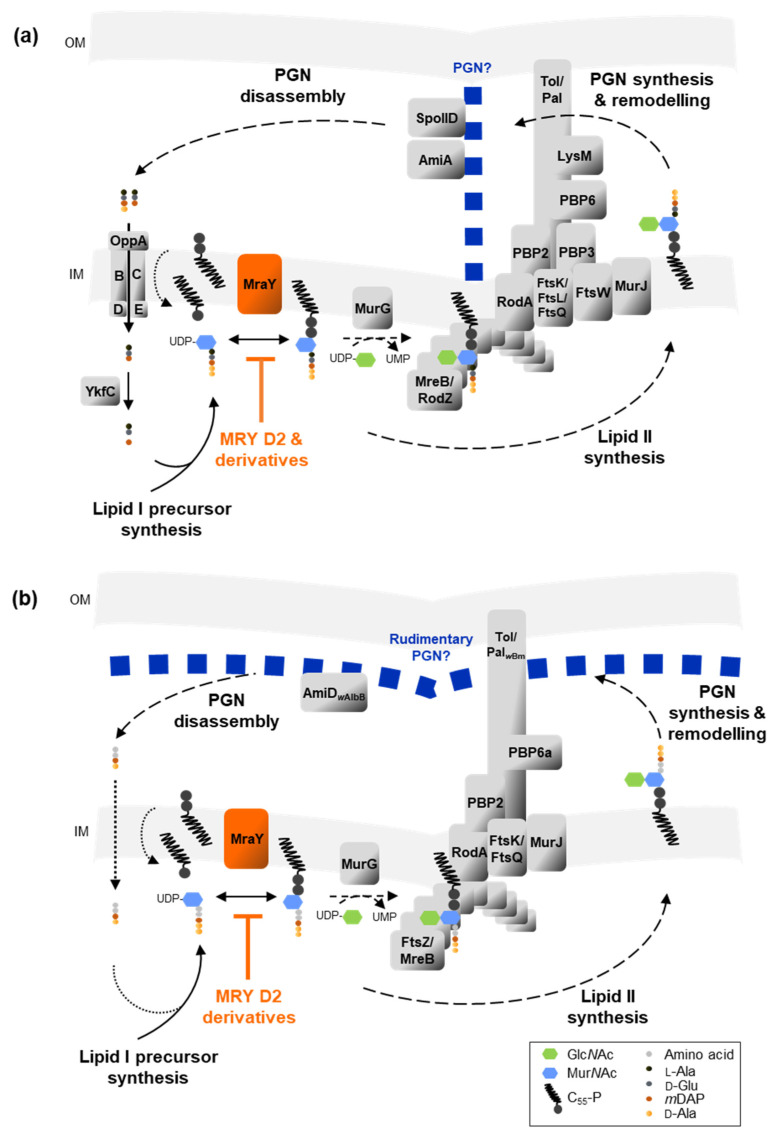
Muraymycins inhibit lipid I formation by binding to MraY and blocking lipid II and peptidoglycan (PGN) synthesis in *C. trachomatis* (**a**) and in *Wolbachia* of *A. albopictus* and *B. malayi* (**b**). Enzymes that are solely conserved in *Wolbachia* of *A. albopictus* (*w*AlbB) or *B. malayi* (*w*Bm) are indicated. Dashed lines indicate inhibited/downregulated processes; dotted lines indicate uncharacterized processes. OM: outer membrane; IM: inner membrane.

**Figure 8:**
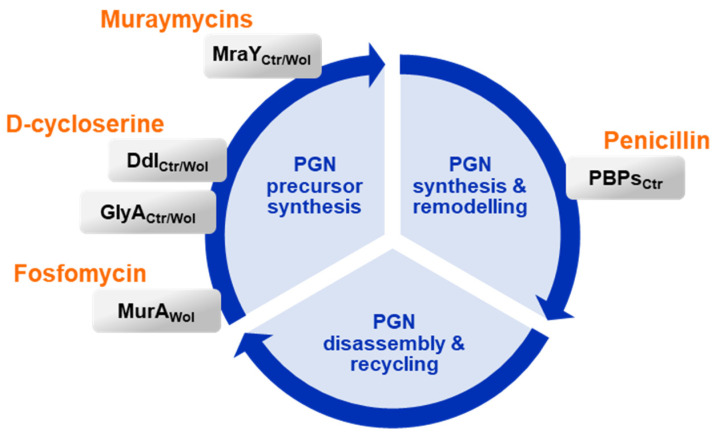
Antibiotic inhibition of enzymes catalyzing important steps in the different PGN synthesis and degradation pathways interrupt the PGN cycle in *C. trachomatis* (Ctr) and *Wolbachia* (Wol), leading to an arrest in cell division.

Most muraymycins tested in this study induced the formation of enlarged chlamydial cells, with one to a few of these ABs per inclusion (Figure 1c and Figure S2). The phenotype is similar to the one seen in PEN G-induced persistent chlamydial infection (Figure 1c) [14]. Compared with MRY D2 inhibiting the formation of lipid I, PEN G acts downstream of MraY in the chlamydial PGN synthesis pathway (Figure 7 and Figure 8). It binds to the transpeptidases PBP2 and PBP3 and the d,d-carboxypeptidase PBP6 [39,64], thereby inhibiting synthesis of cross-linked PGN and blocking cell division [36,37,38]. Although PEN G and MRY D2 both halt chlamydial cell division, different processes are likely to be involved at a cellular level in the bacteria. PEN G-induced persistent chlamydial cells try to compensate for the growth defect by upregulating the transcription of several genes important for PGN precursor synthesis, including the UDP-*N*-acetylglucosamine 1-carboxyvinyltransferase MurA and ligases MurD and MurE. However, the transcription level of MraY is not affected [65]. Furthermore, it was shown by fluorescent labeling of PGN that the PGN ring dissociates under PEN G treatment due to continued PGN degradation accompanied by increased shedding of PGN material [38].

While a few small foci of nascent and non-cross-linked PGN are present under PEN G treatment at an early stage of the chlamydial developmental cycle [38], it is assumed that PGN synthesis is not initiated during the early application of muraymycins. First, transcription of most genes involved in lipid II synthesis starts between >8 hpi to ≤16 hpi [66], which is the time during which the first cell division takes place [14,51]. This includes most Mur enzymes and MraY. Only transcription of the non-canonical alanine racemase GlyA, which converts l-Ala into d-Ala [67], and MurF, which ligates the alanine dipeptide to UDP-Mur*N*Ac-tripeptide [68], starts between >3 hpi and ≤8 hpi [66]. These data indicate that the enzymes for lipid II synthesis, including MraY, are newly synthesized by *Chlamydia* after infection of a new host cell. It would then be inhibited by muraymycins directly or soon after the synthesis of the enzyme. Second, the levels of lipid II are predicted to be very low in Gram-negative bacteria compared with other lipids, as shown for *E. coli*, due to the rapid translocation of lipid II to the periplasm and its incorporation into PGN [69]. Thus, even if there are some molecules of lipid I and II synthesized prior to the inhibition by muraymycins, only a few rounds of cell division might be possible. This would lead to inclusions filled with a few chlamydial cells, as seen for most of the tested muraymycins (Figure 1c and Figure S2) and as shown for PEN G (Figure 1c) [14], compared with untreated inclusions filled with ~ 500 chlamydial cells [14,70]. However, the presence of a few ABs per inclusion could also arise from multiple EBs infecting one host cell. Overall, we propose that upon early inhibition of chlamydial MraY by muraymycins, the production of lipid I and thereby lipid II is abolished and PGN synthesis is not initiated, leading to a block in cell division and subsequent enlargement of chlamydial cells lacking PGN (Figure 7a).

The phenotype induced by PEN G in chlamydial cell culture models is commonly referred to as a persistent state, which is characterized by an inhibition of cell division, enlarged chlamydial cells, an inhibited production of infectious progeny, and the reversibility of this phenotype. Two of these four criteria are fulfilled for muraymycins as the chlamydial cells stop dividing and appear aberrant. Whether or not the muraymycins induce a true persistent state in *Chlamydia*-infected cell culture models, which can be reversed after removal of the antibiotic, resulting in the production of new infectious progeny, needs to be addressed in the future.

As muraymycins had an effect on cell division in intracellular *Chlamydia*, we also investigated the effect against the endosymbiont *Wolbachia*. Although no canonical cell wall has been detected in this genus, a PGN-like structure was visualized using the clickable d-alanine analog ethynyl-d-alanine [31]. We have shown that the endobacteria have retained the ability to form lipid II by MraY and MurG [32], and several other genes involved in PGN synthesis are also conserved [33]. Of these, transcription could be proven for many genes from the *Wolbachia* endosymbiont of *A. albopictus*, including RodA, a protein of the shape, elongation, division, and sporulation (SEDS) family, the transpeptidase PBP2, and the carboxypeptidase PBP6a [33]. In general, *Wolbachia* strains have retained at least one of the SEDS proteins, RodA or FtsW, and at least one monofunctional class B PBP [31,34]. In *Wolbachia* of *B. malayi* and *A. albopictus*, PBP2 and RodA are conserved. Recently, both RodA and FtsW of several Gram-positive bacteria were shown to exhibit monofunctional glycosyltransferase activity [71,72], which would close the gap of the missing glycosyltransferases in *Wolbachia*. Thereby, a minimum of one putative glycosyltransferase and one transpeptidase would be present, which could catalyze the formation of a PGN-like structure in *Wolbachia* [31,34]. Research on cell-wall-targeting antibiotics in *Wolbachia* is helping to elucidate the composition of the PGN synthesis machinery and PGN structure of *Wolbachia* spp.

In this study, the muraymycin derivatives MRH-76 and MRH-92 were tested for their antibacterial activity against *Wolbachia* in cell culture. Both had the same effect: they induced the formation of enlarged *Wolbachia* cells (Figure 2a, b) similarly to muraymycin treatment in *Chlamydia* and resembling the PEN-induced persistent phenotype. Unlike in *Chlamydia*, PEN G does not have an effect on *Wolbachia* [40]; however, the antibiotic fosfomycin, targeting MurA of the lipid II synthesis pathway [73] (Figure 8), also induced the formation of enlarged *Wolbachia* cells [33]. The enlarged cells caused by muraymycins and fosfomycin indicate a block of cell division in *Wolbachia* due to the inhibition of lipid II synthesis [33]. Although the exact PGN structure of *Wolbachia* remains to be described, we propose that PGN synthesis is abolished upon muraymycin treatment (Figure 7b).

Due to the shared similarities between *Chlamydia* and *Wolbachia*, it is conceivable that *Wolbachia* also try to compensate for the growth defect by increasing the transcription of genes involved in PGN precursor synthesis. Furthermore, fewer *Wolbachia* were detected in the host cells compared with untreated infected host cells (Figure 2c). The effect was more pronounced for MRH-76 compared with MRH-92 in regards to the wolbachial median cell diameter and the proportion of enlarged cells. Atwal et al. (2021) observed a similar bacterial depletion compared to the control in the d-cycloserine treatment against *Wolbachia* in *Drosophila melanogaster* [31]. This d-amino acid antibiotic is commonly known to target PGN precursor synthesis by inhibiting alanine racemases and d-alanyl-d-alanine ligases involved in lipid II synthesis (Figure 8). *Wolbachia* has retained and expresses the enzyme Ddl and the alternative d-alanine racemases GlyA and MetC [33], the first two of which are potential targets for d-cycloserine, as shown for *Chlamydia* [67,74]. Whether the depletion of *Wolbachia* by antibiotics targeting lipid II synthesis is an indirect effect of inhibited cell division and less growth over time and/or an active depletion of the bacteria needs to be addressed in the future. However, a similar effect could be expected for muraymycins in vivo in murine models or in patients if the compounds can be absorbed by the tissue, as observed for other *Wolbachia*-depleting antibiotics such as DOX and rifampicin [23,24]. Analyses regarding the reversibility of the phenotype could elucidate whether fosfomycin and muraymycins can induce a persistent-like state in *Wolbachia* as defined for PEN-treated *Chlamydia*.

Since we demonstrated antibacterial activity of muraymycins against *Chlamydia* and *Wolbachia* in cell culture experiments, we next investigated whether this activity is mediated by MraY inhibition. Using inhibition assays performed with recombinant purified MraY_Cpn_, we confirmed the enzymatic inhibition by MRY D2 and MRH-92 (Figure 3b). The enzyme was inhibited at a relatively low concentration compared with the MICs determined for *C. trachomatis*. This difference was also observed for MRY D2, in contrast to its acylated derivative, which had a 32-fold lower MIC for several *S. aureus* strains [45,47]. The lipophilic acyl chain of the derivative increased the overall lipophilicity of the compound and thus the membrane permeability. The MIC of MRH-92 was > 64 µg/mL (Figure 1d). However, both compounds have to traverse four membranes in *C. trachomatis*-infected host cells to reach its target MraY in *Chlamydia*, a process which could contribute to higher MICs in general. Furthermore, the inhibition of MraY_Cpn_ was not altered by the addition of a palmitoyl chain and further derivatization of the peptidic moiety in MRH-92 (Figure 1d and Figure S1), similar to results obtained in inhibition assays with *S. aureus* MraY [48].

For a deeper insight into the enzymatic function and mode of inhibition, we performed a detailed analysis, which included alignments and protein prediction modeling of MraY from *Chlamydia* and *Wolbachia* in comparison to the well-characterized enzyme of *A. aeolicus* [50,54,58]. We included four strains in the analysis: *C. trachomatis*, *C. pneumoniae*, and *Wolbachia* endosymbionts of the human pathogenic filarial nematode *B. malayi* and of the mosquito *A. albopictus*; the latter was used for the cell culture experiments as *Wolbachia* of *B. malayi* is not culturable.

Our analyses showed that MraY_Ctr/Cpn/*w*AlbB/*w*Bm_ were highly conserved with MraY_Aae_ (Table S2). Protein predictions against apoMraY_Aae_ [58] revealed that the transmembrane protein MraY_Ctr/Cpn/*w*AlbB/*w*Bm_ is predicted to have ten transmembrane domains, of which TM3, TM8, and the cytoplasmic loop E form the active site facing the cytoplasm (Figure 4 and Figure 5). In MraY_Aae_, four essential catalytic residues were identified [56,57,58]. While D265 is involved in coordinating the catalytic Mg^2+^ [58], D117 is proposed to bind to the phosphate of the substrate C_55_-P [57,58]. All four catalytic residues of MraY_Aae_ are highly conserved in MraY_Ctr/Cpn/*w*AlbB/*w*Bm_ and are located in the active site (Figure 4 and Figure 5). Due to this, we propose that the endobacterial MraY_Ctr/Cpn/*w*AlbB/*w*Bm_ function like MraY_Aae_, with the respective aspartic acid residues D100/D110/D94/D94 interacting with the substrate C_55_-P and the D243/D256/D229/D229 residues coordinating the Mg^2+^.

Due to the structural similarities of the MraY natural substrate UDP-Mur*N*Ac-pentapeptide and nucleoside antibiotics, the antibiotics are able to bind to and inhibit MraY. One of the best-characterized nucleoside antibiotics is MRY D2, which is competitive for the nucleotide of UDP-Mur*N*Ac-pentapeptide [47,50] and non-competitive for the substrate C_55_-P [47]. Upon binding of MRY D2 to MraY, a conformational change of the enzyme widens the active site, creating a nucleoside- and peptide-binding pocket. The acyl chain of carbacaprazamycin binds in a hydrophobic cleft of MraY_Aae_. Our sequence comparison and 3D modeling of MraY_Ctr/Cpn/*w*AlbB/*w*Bm_ against MraY_Aae_ bound to MRY D2 [50] revealed that the amino acid residues important for binding to the uracil, 5′-aminoribose, and peptidic moiety of MRY D2 and the acyl chain of cCPZ are, with a few exceptions, also highly conserved among these four species and face the cytoplasm or the hydrophobic cleft (Figure 4 and Figure 5). In total, eight amino acids contribute to the binding of the lipophilic side chain in MraY_Aae_. In MraY_Ctr/Cpn/*w*AlbB_, five to seven of these residues are present (Figure 4 and Figure 5). The conserved residues in MraY_Cpn_ are sufficient for the binding of MRH-92 as evidenced by the observed inhibition (Figure 3b). The MRY D2 derivatives are also able to exert antibacterial activity against *C. trachomatis* and *Wolbachia* (Figure 1d and Figure 2). A residue that is not conserved in MraY_Cpn/*w*AlbB_ is A321 of MraY_Aae_, which facilitates binding to the peptidic moiety of MRY D2. This part of the peptidic moiety is not present in the derivatives MRH-38, -76, and -92 (Figure S1); thus, this missing residue in MraY_Cpn_ and MraY*_w_*_AlbB_ is not important for binding these three derivatives. Since MRH-92 and MRY D2 abolished the enzymatic activity of MraY_Cpn_ at the same concentration (Figure 3b), the amino acid exchange from A321 to a serine in MraY_Cpn_ is not detrimental for the inhibitory effect of MRH-92 compared to MRY D2. This also fits with the observation that MRY D2 binding to MraY_Aae_ was most disturbed by D193N and F262A mutations [50], of which both residues are conserved in MraY_Ctr/Cpn/*w*AlbB/*w*Bm_.

Considering the in vitro inhibition of MraY_Cpn_ by muraymycins and the high level of conservation of residues contributing to binding of the antibiotics in MraY_Ctr/Cpn/*w*AlbB/*w*Bm_, we postulate a similar mode of inhibition for muraymycins toward chlamydial and wolbachial MraY as for MraY_Aae_: muraymycins harboring the essential uracil moiety, the 5′-aminoribose, and the 3’-hydroxyl group of the 5′-aminoribose [59,60,61] bind to MraY of *Chlamydia* and *Wolbachia* via the conserved amino acid residues, leading to a conformation change in the enzyme. This binding is competitive for the nucleotide from the natural substrate UDP-Mur*N*Ac-pentapeptide and non-competitive for C_55_-P.

Based on our observations that muraymycins inhibit chlamydial MraY and thereby block further lipid II formation, we were curious about the effects of MRY D2 on a PEN G-induced persistent infection with *C. trachomatis*. Compared with MRY D2, PEN G acts downstream in the PGN cycle by inhibiting PGN transpeptidation and remodeling (Figure 8). A persistent chlamydial state in cell culture models can be induced by β-lactams or stress factors such as nutrient starvation or interferon-γ [13,14]. However, the enlarged chlamydial cell phenotypes can also be detected in vivo in tissue obtained from patients [6,12]. Several studies conducted in vitro showed evidence for β-lactam-induced ABs being less susceptible to treatment with the first-choice antibiotics AZI and DOX [16,17,18]. Reduced susceptibility of persisting chlamydial cells could be one important factor in treatment failures in infected patients, resulting in reoccurring infections with potential long-term effects. Therefore, it is of importance to understand how persistence develops in *Chlamydia* and if persisting *Chlamydia* can be treated in vivo.

First, we tested the simultaneous application of MRY D2 and PEN G at an early development stage, which led to the formation of enlarged chlamydial cells, as seen for MRY D2 alone (Figure 6b and d). No synergistic effects were observed, which might be due to the fact that the effects of MRY D2 occurred upstream of the effects of PEN G activity in PGN biosynthesis. This result is similar to the activity of d-cycloserine. Recently, d-cycloserine was shown to target not only alanine racemases and Ddl but also transpeptidases in *B. subtilis* and *E. coli* due to their structural similarity with the native substrates [75]. For *Chlamydia*, inhibition of the alternative racemase GlyA and MurC/Ddl was shown in vitro [67,74] (Figure 8), and treatment of *C. trachomatis* with d-cycloserine also leads to the formation of enlarged chlamydial cells [29]. Simultaneous application of muraymycins and PEN G interferes with the same PGN synthesis sub-pathway as d-cycloserine, where the effect of inhibiting precursor synthesis outcompetes the inhibition of transpeptidation. However, treatment with d-cycloserine leads to ABs exhibiting nascent, non-cross-linked PGN instead of the lack of a PGN ring, with just a few remaining synthesis nodes of nascent non-cross-linked PGN, as seen with PEN G [38].

MRY D2 treatment of PEN G-induced persisting chlamydial cells cleared 70% of the infection in contrast to cells treated with the β-lactam only (Figure 6b,c). A reduced abundance of inclusions was also observed for MRY D2 treatment alone at the mid-stage of the developmental cycle in an active infection (Figure 6b,c). This indicates that while muraymycins had a bacteriostatic effect when applied early before the EB-to-RB differentiation and cell division, their later application exhibited bactericidal effects on actively dividing or persisting *C. trachomatis* cells.

One reason for the persistence-breaking bactericidal properties of MRY D2 could be the interference with another pathway besides PGN synthesis and/or a secondary target. Tunicamycin, another MraY inhibitor, additionally targets the glycosyltransferases TarO and CapM of the wall teichoic acid pathway and the 2-epimerases MnaA and Cap5P of the capsule synthesis in staphylococci [76,77]. Although these enzymes and MraY catalyze different reactions, they all use an UDP-activated hexose as a substrate. *Chlamydia* do not possess wall teichoic acid, a capsule, or an O-antigen; however, enzymes of other pathways with a similar substrate as MraY might be inhibited, which could then lead to the detrimental effect of the mid-stage application of muraymycins.

Another possible cause for this effect might be the timing itself. Normally, chlamydial cells differentiate into RBs and subsequently start cell division at ~8 hpi to 12 hpi [14,29,51,70]. During cell division, bacteria naturally shed PGN material into their surroundings; in *E. coli*, ~6% to 8% of their PGN is released per generation [78]. In *Chlamydia*, PGN is degraded by the lytic transglycosylase SpoIID, resulting in anhydro-Mur*N*Ac-tetra- or pentapeptides and the amidase AmiA, which releases the peptide chain from the sugar [79,80]. These PGN-derived peptides are subsequently recycled by the cytoplasmic peptidase YkfC by cleaving the γ-d-Glu-*m*DAP bond [81]. PGN fragments containing Mur*N*Ac-l-Ala-γ-d-Glu and l-Ala-γ-d-Glu-*m*DAP motifs are ligands for NOD1 and NOD2 receptors, respectively. These are located in the host cell cytoplasm and exhibit immune modulatory properties via NF-κB activation and the transcription of cytokines [82,83]. Application of muraymycins at the mid-stage of the chlamydial developmental cycle could lead to a complete block of new PGN synthesis that might be accompanied by continued PGN degradation. The antibiotic’s interference with the pathway might lead to an increased shedding of chlamydial PGN, which could trigger the NOD-mediated host response in cervical HEp-2 cells and in cells of the female reproductive tract [84]. This NOD response could contribute to the clearance of chlamydial inclusions by increased inflammation and autophagy [85,86]. Although cell division is nearly completely blocked in PEN-induced persistent cells at 12 hpi [14], some PGN synthesis nodes remain [38]. Furthermore, continued shedding of NOD1 and NOD2 ligands was also reported for *C. trachomatis* upon treatment with different β-lactams [38,87,88]. An additional blockage of PGN precursor synthesis through muraymycins on top of the persistent state could then abolish the remaining PGN synthesis and intensify the cellular stress in these chlamydial cells. This might result in an enhanced NOD-mediated host response, eradicating *Chlamydia* from the host cells.

In contrast to cell culture infection models, *Chlamydia* infections in patients are not synchronized, and the pathogens are present at different stages of their developmental cycle, e.g., the early phase (before cell division started) and the mid-phase (active cell division). This could lead to mixed effects upon muraymycin treatment, inducing aberrance in the early phases and clearance in the mid phases. Cell culture infection models are also limited in reflecting the interplay of multiple stress-inducing factors like iron starvation, interferon-γ induced tryptophan starvation, or viral co-infections that may contribute to persisting phenotypes within infected human tissues and impact the antibiotic effect.

The results presented here support MraY as a promising target for the development of new antibiotic treatment strategies against *Chlamydia* and *Wolbachia*. However, to progress to selecting a muraymycin as a lead candidate for development, further medicinal chemistry is required to improve its potency and solubility, with a focus on penetrating multiple membrane barriers to reach intracellular pathogens and more in-depth analyses of toxicity, pharmacokinetic/pharmacodynamic (PK/PD) determination in appropriate in vivo models, and resistance development.

In conclusion, we showed that MRY D2 and its derivatives interfere with the activity of the transferase MraY in *Chlamydia* and *Wolbachia*. The structural conservation between the MraY of *Chlamydia* and *Wolbachia* and the MraY of *A. aeolicus* suggests that similar binding occurs in a competitive manner toward the natural substrate UDP-Mur*N*Ac. Treatment of *Wolbachia* with muraymycin derivatives resulted in the formation of enlarged bacterial cells, further strengthening the hypothesis that functional PGN synthesis is essential in these symbiotic bacteria of filarial nematodes. The effects of MRY D2 treatment on *Chlamydia* were dependent on the time of application. Based on these observations, we postulate the following: (i) in *Wolbachia* and nondividing *Chlamydia* in their early stage of the developmental cycle, inhibition of MraY-catalyzed lipid I formation has a bacteriostatic effect and induces the formation of enlarged, nondividing cells; (ii) the enlarged, aberrant cells lack PGN; and (iii) the interference of lipid I formation in actively dividing (mid-stage) or persisting *Chlamydia* abolishes (remaining) PGN synthesis and might also interfere with other bacterial or host cell mechanisms, resulting in a bactericidal effect on the *Chlamydia*. The observed persistence-breaking property of MRY D2 could improve the understanding and treatment of reoccurring chlamydial infections in vivo.

## 4. Materials and Methods

### 4.1. Compounds

Muraymycin D2 and its derivatives MRH-22, -23, -25, -38, -76, -82, and -92 [47,48], the caprazamycin analog carbacaprazamycin [49], and DOX (Merck, Darmstadt, Germany) were dissolved in 100% (*v*/*v*) DMSO (Sigma-Aldrich, St. Louis, MO, USA).

### 4.2. Cell Culture and Bacterial Strains

Eukaryotic HEp-2 host cells (ATCC CCL-23, Cell lines service, Eppelheim, Germany) were cultured in Dulbeccos’s Modified Eagle’s Medium (DMEM; Gibco, Carlsbad, CA, USA; item No. 31966021) supplemented with 10% (*v*/*v*) fetal calf serum (FCS; Gibco; item no. 10270106), 1 × MEM nonessential amino acids solution (Gibco), 1 × MEM vitamin solution (Gibco), 2.5 µg/mL amphotericin B (Gibco), and 50 µg/mL gentamicin (Gibco) at 37 °C and 5% (*v*/*v*) CO_2_. Passaging of *C. trachomatis* D/UW-3/CX (ATCC VR-885) was performed every 2 to 3 days. Briefly, EBs were harvested by scraping off the host cell monolayer, vortexed with glass beads for 30 s, and centrifuged at 1480× *g* and 25 °C for 10 min. HEp-2 monolayers were inoculated with the EB suspension diluted in medium and incubated for 3 h. Then, the suspension was exchanged for fresh medium containing 1.2 µg/mL cycloheximide (Sigma-Aldrich). Cell cultures were tested monthly for *Mycoplasma* contamination using the Venor GeM OneStep Mycoplasma Detection Kit (Minerva Biolabs, Berlin, Germany). All cell culture experiments were conducted in supplemented growth DMEM medium without gentamicin, amphotericin B, and cycloheximide.

The insect cell line *A. albopictus* C6/36 (ATCC CRL-1660) was used to propagate *Wolbachia* endosymbionts (*Wolbachia* strain B of *A. albopictus*, as described in [32,33,89]. The cells were incubated in L15 Leibovitz’s medium (Life Technologies, Carlsbad, CA, USA) supplemented with 5% (*v*/*v*) FCS (PAA Laboratories, Cölbe, Germany), 1% (*v*/*v*) nonessential amino acids (PAA Laboratories), 2% (*w*/*v*) tryptose phosphate broth (Sigma-Aldrich), and 1% (*v*/*v*) penicillin/streptomycin (PAA Laboratories, Cölbe, Germany) at 26 °C.

### 4.3. Cytotoxicity of Compounds toward HEp-2 Cells

Cytotoxicity of the compounds toward HEp-2 cells was assessed using the resazurin-based alamarBlue cell viability reagent (Thermo Fisher Scientific, Waltham, MA, USA). 5 × 10^4^ HEp-2 cells/mL were seeded into flat base TC-96-well plates (Sarstedt, Nümbrecht, Germany) and incubated at 37 °C and 5% (*v*/*v*) CO_2_. After 48 h, the cells were washed with medium, followed by the addition of compounds in a serial dilution ranging from 128 µg/mL to 1 µg/mL for another 28 h. Next, the cells were washed twice with Hank’s balanced salt solution (HBSS) and incubated with the alamarBlue cell viability reagent diluted 1:10 in HBSS for 1 h. The conversion of resazurin into resorufin by viable cells was determined by transferring the supernatant into black 96-well plates (Greiner Bio-One, Frickenhausen, Germany) and measuring the fluorescence at 550 nm excitation and 595 nm emission wavelengths using a Tecan infinite M200 plate reader and Tecan SparkControl software v3.2 (Tecan Group, Männedorf, Switzerland). HEp-2 cell viability was normalized to the vehicle control, and the cytotoxic concentration 50% (CC_50_) was calculated by non-linear regression using GraphPad Prism v10.1.2 (GraphPad Software, San Diego, CA, USA).

### 4.4. Effect of Compounds on Active and Penicillin G-Induced Persistent C. trachomatis Infections

The effect of the compounds on active *C. trachomatis* infections was analyzed by fluorescence microscopy. First, 3.5 × 10^4^ HEp-2 cells/mL were seeded into black 96-well plates (ibidi, Gräfeling, Germany) and incubated for 3 days at 37 °C and 5% CO_2_. The cells were washed once with medium and infected with freshly prepared *C. trachomatis* D/UW-3/CX EB-containing suspension. After 2 h incubation, the EB suspension was aspirated, and the cells were washed twice with medium. The effect of compounds on an infection was determined by application of the compound at 2 hpi or 12 hpi in a serial dilution from 1 µg/mL to 64 µg/mL (MRH-25, -76, -92, cCPZ) or from 1 µg/mL to 128 µg/mL (MRY D2, MRH-22, -23, -38, and -82). Negative controls were conducted with DMSO as a vehicle control. For *Chlamydia*, the MIC is defined as a >90% reduction in inclusions [52]. The MIC of compounds inducing the formation of enlarged, aberrant bodies is the lowest concentration that induces the abnormal inclusion morphology [39].

The effect of MRY D2 on penicillin-induced persistent *C. trachomatis* infections was analyzed as described for the active infection, with the following modifications. After removal of the EB-containing lysate at 2 hpi and washing of the cells, 100 U/mL PEN G was added to induce a persistent infection. MRY D2 was added at 12 hpi in a concentration range of 128 µg/mL to 1 µg/mL by serial dilution. In another approach, MRY D2 was applied simultaneously with PEN G at 2 hpi to determine if early administration influences the establishment of a persistent infection.

Infected HEp-2 cells were fixed and permeabilized with 100% (*v*/*v*) ice-cold methanol at 30 hpi and washed once with 1 × phosphate-buffered saline (PBS, 4 mM KH_2_PO_4_ pH 7.4, 16 mM Na_2_HPO_4_, 115 mM NaCl). The Pathfinder Chlamydia Culture Confirmation System (Bio-Rad Laboratories, Hercules, CA) was used to stain the *C. trachomatis* lipopolysaccharide and host cell cytoplasm by incubation for 30 min at 37 °C. Genomic DNA was stained with 10 µg/mL DAPI (Thermo Fisher Scientific) for 1 min and then washed twice with PBS for 10 min at 4 °C. Fluorescence microscopy was performed with an Axio observer Z.1 using the Zen2 software (Carl Zeiss, Oberkochen, Germany).

Inclusions were quantified using CellProfiler 4.0.6. Cell image analysis software (Borad Institute, Cambridge, MA, USA; [90]) was used to identify fluorescein-labeled *Chlamydia* clusters as the primary objects. The chlamydial cell phenotype in each inclusion was assessed manually and categorized by inclusions filled with (i) EBs and/or RBs, (ii) enlarged, aberrant bodies similar to the penicillin G-induced ABs, and (iii) a mixed phenotype of inclusions filled with EBs/RBs and AB (typically just one). Statistical analysis for all parameters was performed with an unpaired, two-tailed Student’s *t*-test against the respective vehicle control using GraphPad Prism software version 10.1.2 for Windows (GraphPad Software).

### 4.5. Fluorescence Microscopy of Muraymycin-Treated Wolbachia

The effect of muraymycin derivatives on the phenotype of *Wolbachia* was analyzed by fluorescence microscopy as described [33], with minor modifications. Briefly, 1.5 × 10^4^ *A. albopictus* C6/36 cells infected with *Wolbachia* were seeded into 8-well culture slides and incubated with or without compound at 26 °C. Medium was exchanged every third day, with and without compound. The cells were fixed on day 9 using 4% (*w*/*v*) paraformaldehyde in PBS. The cells were permeabilized with 0.25% (*v*/*v*) Triton X-100 and washed with PBS supplemented with 2% (*w*/*v*) bovine serum albumin (BSA Fraction V, Fisher Scientific, Schwerte, Germany) and 0.1% (*v*/*v*) Triton X-100 (PBST). Genomic DNA was stained with 0.25 µg/mL DAPI (Sigma-Aldrich), and the cells were washed with PBST. Slides were embedded in Vectashield Mounting Medium (Vector Laboratories, Newark, CA, USA), and microscopy was performed with a Leica fluorescence microscope DM RD (Leica Camera, Wetzlar, Germany). Photoshop CS2 software (Adobe Systems, San Jose, CA, USA) was used for editing the sharpness, brightness, and contrast of the images. ZEN 3.5 (blue edition) software (Zeiss, Oberkochen, Germany) was used in combination with the image analysis module to determine the diameter of *Wolbachia*. Enlarged cells were defined as having a diameter greater than the 75th percentile of the DMSO control. For statistical analysis, a Kruskal–Wallis test was performed using GraphPad Prism software version 10.1.2 for Windows (GraphPad Software).

### 4.6. Quantification of Wolbachia

Susceptibility of *Wolbachia* toward different compounds was tested as described [32,53]. In short, 1 × 10^4^ cells/well of *A. albopictus* C6/36 infected with W*olbachia* were cultured in supplemented medium in 96-well plates with or without compound at 26 °C for 9 days. The medium, with and without compound, was exchanged every third day, and the cells were harvested on day 9 for the extraction of genomic DNA using the QIAamp Kit (Qiagen, Hilden, Germany) according to the manufacturer’s protocol. *Wolbachia* were quantified by qPCR of the *16S rDNA* gene of *Wolbachia* (GenBank accession No. X61767) and the *actin B* gene of *A. albopictus* (GenBank accession no. DQ657949) as described [32,91]. A plasmid containing the appropriate insert was used to generate a standard curve to determine the copy number of the genes. The abundance of *Wolbachia* was normalized by calculating the ratio of *16S rDNA* copies/µL to *actin B* copies/µL.

### 4.7. Purification of C. pneumoniae MraY

The heterologous transmembrane protein *C. pneumoniae* MraY (MraY_Cpn_, primary accession No. A0A0F7WR30) was produced as described in Henrichfreise et al. (2009) [32], with minor changes. Briefly, the plasmid pET20+*mraY_Cpn_* was transformed into *E. coli* C43(DE3) [92], and expression of transcription was induced in 4 L 2 × YT medium (16 g/L tryptone, 10 g/L yeast extract, 5 g/L NaCl, pH 7.0) by 1 mM IPTG (Thermo Fisher Scientific) at an OD_600nm_ of 0.6 with shaking at 25 °C for 16 h. Harvested cells were washed in 200 mL of 25 mM Tris-HCl (pH 8) and resuspended in buffer A (25 mM Tris-HCl (pH 8.0), 2 mM 2-mercaptoethanol, 150 mM NaCl, 30% (*v*/*v*) glycerol, 1 mM MgCl_2_). After adding 1 U/mL benzonase (Sigma-Aldrich) and increasing the MgCl_2_ concentration to 3 mM, the cells were sonicated and centrifuged at 31.85× *g* at 4 °C for 30 min. The pellet was resuspended in 10 mL buffer A supplemented with 17.8 mM n-dodecyl-β-D-maltoside (DDM, Carl Roth, Karlsruhe, Germany) and incubated for 50 min at 4 °C. The supernatant was separated from membrane debris by centrifugation, and the obtained pellet was solubilized a second time with 21.5 mM DDM. Each supernatant containing MraY_Cpn_ was then incubated with 1.5 mL Ni-NTA agarose (Macherey-Nagel, Düren, Germany) and equilibrated twice with 10 mL buffer A under gentle shaking at 4 °C for 3 h. The mixtures were poured onto affinity chromatography columns and rinsed with 7.2 mL buffer A followed by 7.2 mL washing buffer (buffer A supplemented with 3.9 mM DDM, 10 mM imidazole). MraY_Cpn_ was eluted by the sequential addition of 3 × 0.5 mL elution buffer (buffer A with 3.9 mM DDM) containing 100 mM, 200 mM, and 300 mM imidazole, respectively. Eluates were stored at −70 °C.

### 4.8. Inhibition of C. pneumoniae MraY In Vitro

Inhibition assays with MraY_Cpn_ and the compounds were performed based on the activity assays for MraY_Cpn_ as previously described [32], with minor modifications, in a final volume of 50 µL. First, 2.5 nmol C_55_-P (Larodan, Solna, Sweden) was dissolved in 0.43% (*v*/*v*) Triton X-100 by vortexing for 1 min. Then, 75 mM Tris-HCl (pH 7.5), 6 mM MgCl_2_, 10% (*v*/*v*) DMSO, and UDP-Mur*N*Ac-pentapeptide obtained from *Bacillus cereus* DSM2302 (based on [93]) were added. Compounds were applied at the indicated concentrations and incubated with 2 µg MraY_Cpn_ at 30 °C for 90 min. The reaction products were extracted with 50 µL *n*-butanol-pyridine acetate (2:1, pH 4.2) by vortexing for 1 min. After centrifugation at 17,000× *g* for 5 min, components of the organic phase were separated by thin layer chromatography (TLC) on HPTLC alumina silica gel 60 plates (Merck) using chloroform–methanol–water–ammonia (88:48:10:1) as a mobile phase [94]. The TLC plates were stained with phosphomolybdic acid stain (2.5% (*w*/*v*) phosphomolybdic acid, 1% (*w*/*v*) ceric-sulphate, 6% (*v*/*v*) sulfuric acid) and visualized at 120 °C. The experiments were performed in duplicate.

### 4.9. In Silico Analysis of MraY

The MraY protein sequence of *A. aeolicus* VF5 (primary accession No. O66465), *C. trachomatis* D/UW-3/CX (primary accession No. O84762), *C. pneumoniae* GiD (primary accession No. A0A0F7WR30), and *Wolbachia* endosymbionts of *A. albopictus* (primary accession No. A0A4S2QUK2) and *B. malayi* (primary accession No. Q5GRZ3) were analyzed in silico. Multiple alignments were performed with Clustal Omega Software v1.2.4 (EMBL-EBI, Hinxton, UK). Predictions of MraY_Ctr/Cpn/*w*AlbB/*w*Bm_ protein models were made with one-to-one threading against the crystal structure of the apoMraY_Aae_ chain A (PDB ID: 4J72.A, [58]) or of MraY_Aae_ bound to MRY D2 (PDB ID: 5CKR, [50]) using phyre2 software (Imperial College London, London, UK; [95]) and visualized with ChimeraX (UCSF, San Francisco, CA, USA; [96]). Residues of the MraY_Aae_ active site or residues contributing to the binding of muraymycin D2 or carbacaprazamycin were indicated in the multiple alignment and protein models based on [54,55,56,57,58].

## Data Availability

The original contributions presented in the study are included in the article/Supplementary Material. Further inquiries can be directed to the corresponding authors.

## Supplementary Materials

The following supporting information can be downloaded at: https://www.mdpi.com/article/10.3390/antibiotics13050421/s1, Table S1: Cytotoxic concentration 50% (CC_50_) of muraymycin (MRY) D2, its derivatives (MRHs), and carbacaprazamycin (cCPZ) on eukaryotic HEp-2 cells, Figure S1: Chemical structures of muraymycin derivatives and carbacaprazamycin, Figure S2: Most muraymycin derivatives induce the formation of enlarged chlamydial cells, Table S2: Sequence identities and confidence of *C. trachomatis*, *C. pneumoniae*, and *Wolbachia* endosymbionts of *A. albopictus* or *B. malayi* MraY protein models against *A. aeolicus* apoMraY or MraY_Aae_ bound to muraymycin D2.
