# Supplementary material for: The MraY Inhibitor Muraymycin D2 and Its Derivatives Induce Enlarged Cells in Obligate Intracellular Chlamydia and Wolbachia and Break the Persistence Phenotype in Chlamydia
Source: Antibiotics (Basel). 2024 May 4;13(5):421. doi: 10.3390/antibiotics13050421 (PMC11117252; doi:10.3390/antibiotics13050421)
Supplement: Supplementary file 1 [file antibiotics-13-00421-s001.zip › antibiotics-2923908-supplementary.pdf]

antibiotics

MDPI

Supplementary Material

The MraY Inhibitor Muraymycin D2 and Its Derivatives Induce Enlarged Cells in Obligate Intracellular *Chlamydia* and *Wolbachia* and Break the Persistence Phenotype in *Chlamydia*

Iris Löckener [1,*,†], Lara Vanessa Behrmann [2,†], Jula Reuter [1,†], Andrea Schiefer [2], Anna Klöckner [1], Sebastian Krannich [1], Christian Otten [1], Katja Mölleken [3], Satoshi Ichikawa [4], Achim Hoerauf [2,5], Tanja Schneider [1,5], Kenneth M Pfarr [2,5,*,‡] and Beate Henrichfreise [1,‡]

[1] Institute for Pharmaceutical Microbiology (IPM), University of Bonn, University Hospital Bonn, Meckenheimer Allee 168, 53115 Bonn, Germany
[2] Institute for Medical Microbiology, Immunology and Parasitology (IMMIP), University Hospital Bonn, Venusberg-Campus 1, 53127 Bonn, Germany
[3] Institute for Functional Microbial Genomics, Heinrich Heine University Düsseldorf, Universitätsstraße 1, 40225 Düsseldorf, Germany
[4] Faculty of Pharmaceutical Sciences, Hokkaido University, Kita-12, Nishi-6, Kita-ku, Sapporo 060-0812, Japan
[5] German Center for Infection Research (DZIF), Partner Site Bonn-Cologne, 53127 Bonn, Germany
* Correspondence: iris.loeckener@uni-bonn.de (I.L.); kenneth.pfarr@ukbonn.de (K.P.); Tel.: +49-228-73-4637 (I.L.); +49-288-287-11207 (K.P.)
† These authors contributed equally to this work.
‡ Shared senior authorship.

Table S1. Cytotoxic concentration 50% (CC_{50}) of muraymycin (MRY) D2, its derivatives (MRHs), and carbacaprazamycin (cCPZ) on eukaryotic HEp-2 cells. Muraymycin D2, its derivatives, and cCPZ were incubated with HEp-2 cells for 28 h. The cytotoxicity was measured via alamarBlue normalized against dimethyl sulfoxide and the CC_{50} was calculated by non-linear regression from the mean of two independent experiments. The CC_{50} of muraymycin D2 and the derivatives MRH-22, -23, -38, and -82 was greater than the highest concentration tested (128 μg/mL). MRH-25, -76, -92, and cCPZ were more toxic with CC_{50} values between 81 μg/mL and 124 μg/mL.

**Compound**	**CC_{50} (μg/mL)**
MRY D2	> 128
MRH-22	> 128
MRH-23	> 128
MRH-25	116
MRH-38	> 128
MRH-76	81
MRH-82	> 128
MRH-92	109
cCPZ	124

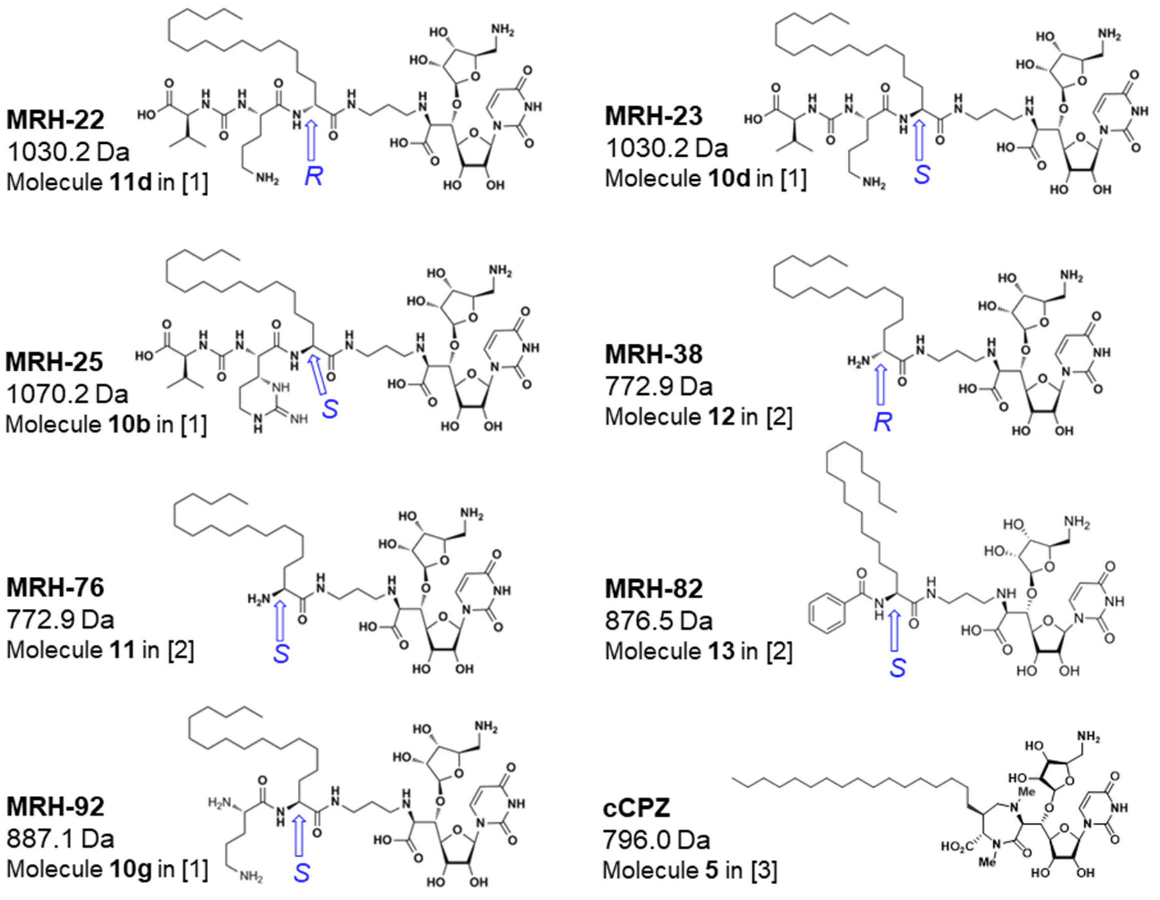

Figure S1. Chemical structures of muraymycin derivatives and cCPZ. All compounds were previously described [1–3]. The core structure of muraymycins resembles uridine diphosphate *N*-acetylmuramoyl-pentapeptide, the last cytoplasmic precursor molecule of lipid I. Muraymycin derivatives and cCPZ are more lipophilic due to an additional palmitoyl side chain. Important chemical configurations of muraymycin derivatives are indicated by a blue arrow.

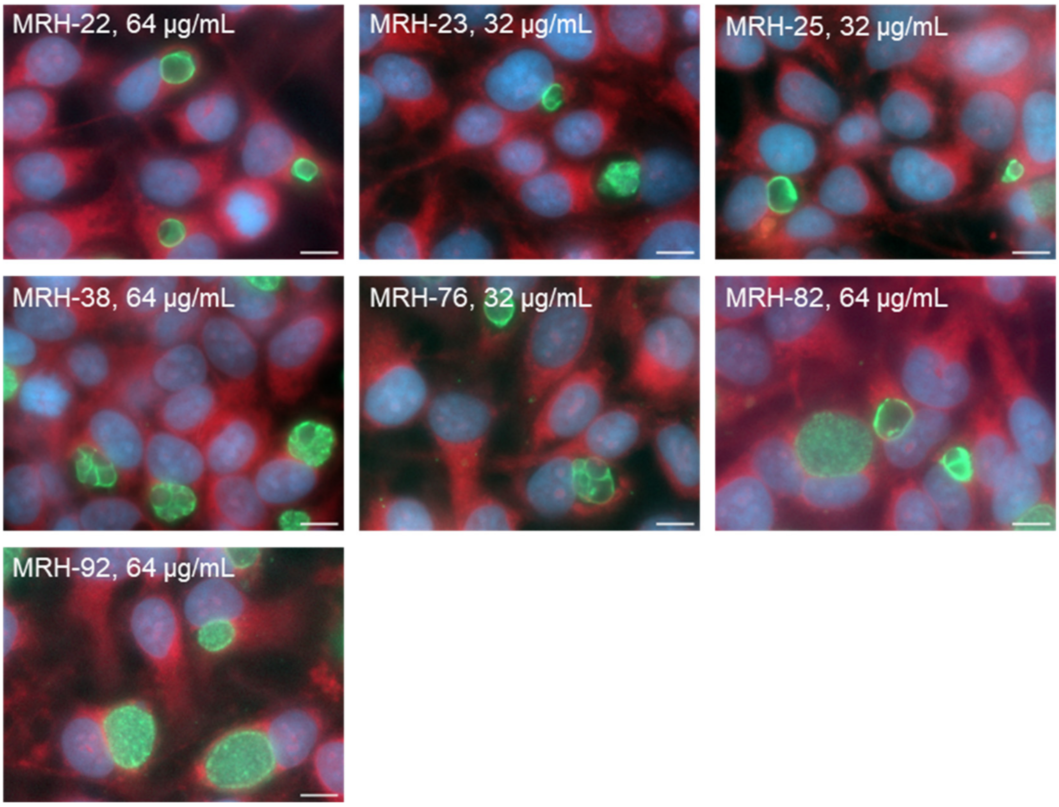

Figure S2. Most muraymycin derivatives induce the formation of enlarged chlamydial cells. HEp-2 cells infected with *C. trachomatis* D/UW-3/CX were treated with muraymycin derivatives at 2 h post infection (hpi). At 30 hpi, the anti-chlamydial activity was analyzed for concentrations which did not exert cytotoxicity towards the host cells in resazurin-based viability assays (Table S1) and in fluorescence microscopy analyses. Eukaryotic host cell cytoplasm from representative images was labelled with Evans Blue (red), DNA with DAPI (blue), and chlamydial lipopolysaccharide with fluorescein (green). Scale bar: 10 µm. All derivatives tested are shown at their minimal inhibitory concentration, which is reflected by the presence of aberrant bodies. MRH-92 did not induce a persistence-like phenotype and is shown at the highest concentration tested (64 µg/mL).

Table S2. Sequence identities and confidence of *C. trachomatis*, *C. pneumoniae*, and *Wolbachia* endosymbionts of *A. albopictus* or *B. malayi* MraY protein models against *A. aeolicus* apoMraY or $MraY_{Aae}$ bound to muraymycin D2. Bacterial strains and primary protein accession numbers: *A. aeolicus* VF5 (Aae, O66465), *C. trachomatis* D/UW-3/CX (Ctr, O84762), *C. pneumoniae* GiD (Cpn, A0A0F7WR30), and *Wolbachia* endosymbiont of *A. albopictus* (*w*AlbB, A0A4S2QUK2) and *B. malayi* (*w*Bm, Q5GRZ3). Predictions of $MraY_{Cpn/Ctr/wAlbB/wBm}$ protein models were made with one-to-one threading against crystal structures of $apoMraY_{Aae}$ chain A [4] or $MraY_{Aae}$ bound to MRY D2 [5] using phyre2 software (Imperial College London, London, UK; [6]).

Query	$MraY_{Aae}$ template	Sequence identity (%)	Aligned residues	Confidence (%)
$MraY_{Ctr}$	$apoMraY_{Aae}$ chain A	33	306/336 (= 91 %)	100
	$MraY_{Aae}$ bound to MRY D2	33	311/336 (= 93 %)	100
$MraY_{Cpn}$	$apoMraY_{Aae}$ chain A	37	308/349 (= 88 %)	100
	$MraY_{Aae}$ bound to MRY D2	37	316/349 (= 91 %)	100
$MraY_{wAlbB}$	$apoMraY_{Aae}$ chain A	41	301/326 (= 92 %)	100
	$MraY_{Aae}$ bound to MRY D2	42	305/326 (= 94 %)	100
$MraY_{wBm}$	$apoMraY_{Aae}$ chain A	40	302/326 (= 93 %)	100
	$MraY_{Aae}$ bound to MRY D2	40	303/326 (= 93 %)	100

References

1. Tanino, T.; Al-Dabbagh, B.; Mengin-Lecreulx, D.; Bouhss, A.; Oyama, H.; Ichikawa, S.; Matsuda, A. Mechanistic Analysis of Muraymycin Analogues: A Guide to the Design of MraY Inhibitors. *J Med Chem* **2011**, *54*, 8421–8439, doi:10.1021/jm200906r.
2. Takeoka, Y.; Tanino, T.; Sekiguchi, M.; Yonezawa, S.; Sakagami, M.; Takahashi, F.; Togame, H.; Tanaka, Y.; Takemoto, H.; Ichikawa, S.; et al. Expansion of Antibacterial Spectrum of Muraymycins toward *Pseudomonas Aeruginosa*. *ACS Med Chem Lett* **2014**, *5*, 556–560, doi:10.1021/ml5000096.
3. Ichikawa, S.; Yamaguchi, M.; Hsuan, L.S.; Kato, Y.; Matsuda, A. Carbacaprazamycins: Chemically Stable Analogues of the Caprazamycin Nucleoside Antibiotics. *ACS Infect Dis* **2015**, *1*, 151–156, doi:10.1021/id5000376.
4. Chung, B.C.; Zhao, J.; Gillespie, R.A.; Kwon, D.-Y.; Guan, Z.; Hong, J.; Zhou, P.; Lee, S.-Y. Crystal Structure of MraY, an Essential Membrane Enzyme for Bacterial Cell Wall Synthesis. *Science* **2013**, *341*, 1012–1016, doi:10.1126/science.1236501.
5. Chung, B.C.; Mashalidis, E.H.; Tanino, T.; Kim, M.; Matsuda, A.; Hong, J.; Ichikawa, S.; Lee, S.-Y. Structural Insights into Inhibition of Lipid I Production in Bacterial Cell Wall Synthesis. *Nature* **2016**, *533*, 557–560, doi:10.1038/nature17636.
6. Kelley, L.A.; Mezulis, S.; Yates, C.M.; Wass, M.N.; Sternberg, M.J.E. The Phyre2 Web Portal for Protein Modeling, Prediction and Analysis. *Nat Protoc* **2015**, *10*, 845–858, doi:10.1038/nprot.2015.053.
